# Landscape of Steroid Dynamics in Pregnancy: Insights From the Maternal-Placental-Fetal Unit and Placental Models

**DOI:** 10.1016/j.mcpro.2025.100976

**Published:** 2025-04-23

**Authors:** Rona Karahoda, Therina Du Toit, Barbara Fuenzalida, Sampada Kallol, Michael Groessl, Pascale Anderle, Edgar Ontsouka, Frantisek Staud, Christa E. Flueck, Christiane Albrecht

**Affiliations:** 1 Department of Pharmacology and Toxicology, Charles University, Faculty of Pharmacy in Hradec Kralove, Hradec Kralove, Czech Republic; 2 Department of Biomedical Research, Inselspital, University of Bern, Bern, Switzerland; 3 Institute of Biochemistry and Molecular Medicine, University of Bern, Bern, Switzerland; 4 Department of Nephrology and Hypertension, University Hospital Bern, Bern, Switzerland; 5 Foundation HSeT, Epalinges, Switzerland; 6 Division of Pediatric Endocrinology, Diabetology and Metabolism, Department of Pediatrics, Inselspital, Bern University Hospital, University of Bern, Bern, Switzerland

**Keywords:** steroidogenesis, placenta, pregnancy, androgen, progesterone

## Abstract

Recent advances in analytical methods have revolutionized our understanding of steroid biochemistry. The emergence of novel steroids such as 11-oxy androgens and 11-oxy progesterones has necessitated a reevaluation of steroid biosynthesis and metabolism within the maternal-placental-fetal unit. In this study, we employed a validated liquid chromatography high-resolution mass spectrometry method to quantify 51 steroids in paired maternal serum, neonatal serum, and placenta samples from 37 healthy pregnancies. Additionally, we characterized steroid release in various placental models, including human placenta perfusion, explants, and primary trophoblast cells isolated from human term placenta. Our findings emphasize the predominance of keto derivatives of androgens in the placenta compared to hydroxylated forms, which are dominant in maternal serum and neonatal serum. We also observed high levels of classic and novel progesterones in the placenta and across all models, with significant release on the maternal side. These results suggest that the placenta possesses an active enzymatic machinery capable of producing and metabolizing novel progesterones. Furthermore, we demonstrated that the catalytic activity of 11β-hydroxysteroid dehydrogenase type 2 extends beyond cortisol regulation to hydroxylated androgens, highlighting its significance in the broader context of steroid metabolism within the maternal-placental-fetal unit. These findings contribute to our understanding of placental physiology and impact on fetal development.

## Highlights

• Profiling of 51 steroids in maternal/fetal serum, placenta, and placental models.
• Keto-androgens predominate in the placenta; serum is rich in hydroxylated forms.
• Placenta actively produces and releases classic and novel progesterones.
• 11βHSD2 enzymatic activity extends to hydroxylated androgens.
• Placenta plays a key role in novel steroid metabolism for fetal development.

## Graphical Abstract

**Figure:**
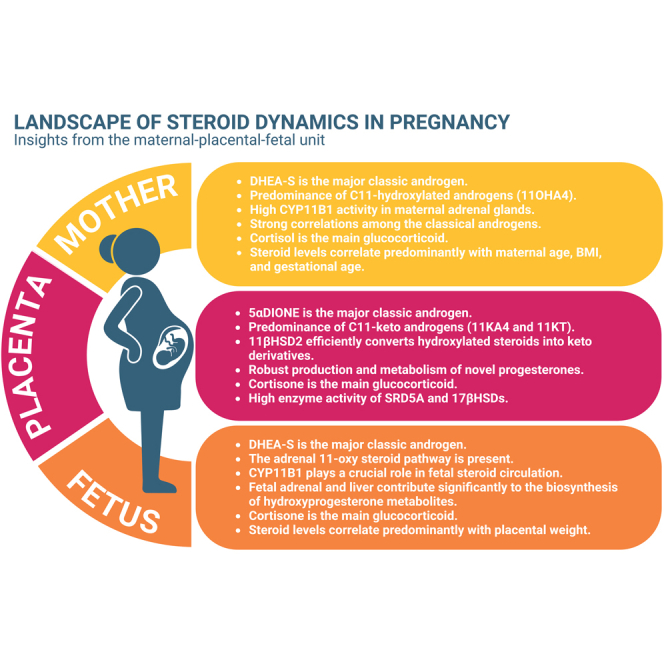

## In Brief

This study provides a comprehensive characterization of steroid metabolism in the maternal-placental-fetal unit by profiling 51 steroids in maternal, neonatal, and placental samples, complemented by multiple placental models. The findings reveal that the placenta favors keto derivatives of androgens and actively produces classic and novel progesterones. The study also broadens the role of placental 11βHSD2 beyond cortisol metabolism, offering new insights into placental steroidogenesis and its relevance for fetal development.

Steroid hormone synthesis and secretion are fundamental for maintaining pregnancy and modulating maternal and fetal physiology throughout gestation (1). Derived from the common precursor cholesterol, steroid hormones comprise sex steroids (progestogens, androgens, estrogens) and corticosteroids (glucocorticoids and mineralocorticoids). These hormones play numerous critical roles during pregnancy, including promoting immunotolerance, regulating metabolic homeostasis, and supporting decidualization, trophoblast proliferation, and invasion (2, 3, 4, 5). They also contribute to developing sexual characteristics, maternal vasculature and endothelial cell proliferation modulation, embryo implantation, angiogenesis, and overall fetal growth (6, 7).

During pregnancy, steroid concentrations in the bloodstream are influenced by secretory activities of steroidogenic tissues, including adrenals, testes, ovaries, and the placenta. Sharing the ability for *de novo* steroid synthesis, these steroidogenic tissues are collectively referred to as the maternal-placental-fetal unit. Pivotal in this unit, the placenta further regulates the steroid hormone exchange between the mother and the fetus through its metabolic and transport function (8). Despite their importance, our understanding of steroid hormone dynamics in pregnancy is limited. Recent advances in liquid chromatography-mass spectrometry have enabled identifying and quantifying a broader range of classic and novel steroids, termed C11-oxy, 11-oxygenated, or 11-oxy steroids, such as 11-oxy androgens and 11-oxy progesterones (9). Some of these 11-oxy steroids, including 11β-hydroxyandrostenedione (11OHA4) and its metabolites, 11-ketoandrostenedione (11KA4) and 11-ketotestosterone (11KT), have been identified in the maternal-placental-fetal unit (10, 11, 12, 13, 14, 15). Current evidence indicates that 11-oxy androgens originate from the adrenal glands, where they are biosynthesized from androstenedione (A4) through C11 hydroxylation by cytochrome P450 11β-hydroxylase (CYP11B1) (16, 17, 18, 19). The metabolism of 11OHA4 by 11β-hydroxysteroid dehydrogenase type 2 (11βHSD2) results in C11-keto derivatives (19, 20), potent androgens that are trafficked between the fetus and placenta.

Although the placenta is not typically considered an androgen-producing organ, its role in androgen metabolism is important due to several factors. Firstly, the placenta expresses the enzyme cytochrome P450 aromatase (CYP19A1), which converts androgens into estrogens, a process crucial for maintaining estrogen levels necessary for pregnancy maintenance and fetal development (21, 22, 23). Secondly, the placenta expresses both the oxidative and reductive forms of 17β-hydroxysteroid dehydrogenases (24, 25, 26), indicating involvement in androgen metabolism, particularly in the inactivation of testosterone (T) to A4. Thirdly, the high expression of 11βHSD2 in the placenta is notable (22). While historically recognized for deactivating cortisol (F) to protect the fetus from excess maternal glucocorticoids (27), 11βHSD2 also metabolizes 11-oxy steroids, converting them into their active forms (10, 28, 29), suggesting significant contributions to both glucocorticoid and androgen metabolism. Fourthly, the placenta expresses steroid 5α-reductase (SRD5A), an enzyme that converts T to dihydrotestosterone (DHT). However, SRD5A is also implicated in the reduction of 11-keto steroids (30), indicating its potential role in local classic and novel androgen metabolism. Lastly, the potential backdoor pathway for androgen production in the placenta, which bypasses the conventional intermediates in androgen biosynthesis, highlights the complex regulatory mechanisms of steroidogenesis in the placenta (6).

In addition to 11-oxy androgens, 11-oxy progesterones, such as 21-deoxycortisol (21dF) and 11-hydroxyprogesterone (11OHP4), are of interest. These steroids, detected in neonates (12, 31, 32), can be metabolized to 11-oxy androgen precursors, potentially contributing to the pool of active androgens (33, 34). Moreover, 17α-hydroxyprogesterone (17OHP4) can contribute to the androgen pool via the backdoor pathway, leading to the production of the active androgen DHT (35). Importantly, 11βHSD2 may also metabolize 11OHP4 to yield 11-ketoprogesterone (11KP4) (36). Considering the important role of progesterone (P4) in maintaining uterine quiescence, 11βHSD2 may potentially further contribute to events associated with labor onset.

In light of this new evidence (10, 11, 30), reexamining the steroid biosynthetic capacity in the maternal-placental-fetal unit is necessary. While the physiological roles and regulatory mechanisms of many novel steroids remain largely unexplored, their potential as biomarkers for pregnancy-related disorders and their influence on fetal and maternal health are areas of active investigation (28, 29). It must be stressed that the dynamic interactions between maternal, fetal, and placental compartments complicate the study of steroid hormones. Our current understanding of the steroidogenic landscape in the maternal-placental-fetal unit is based predominantly on serum measurements (11, 12); while this provides systemic information, it may miss localized steroid (dys)regulations. Thus, investigating the placental steroid microenvironment allows additional insight into local steroid metabolism (10).

**Fig. 1:**
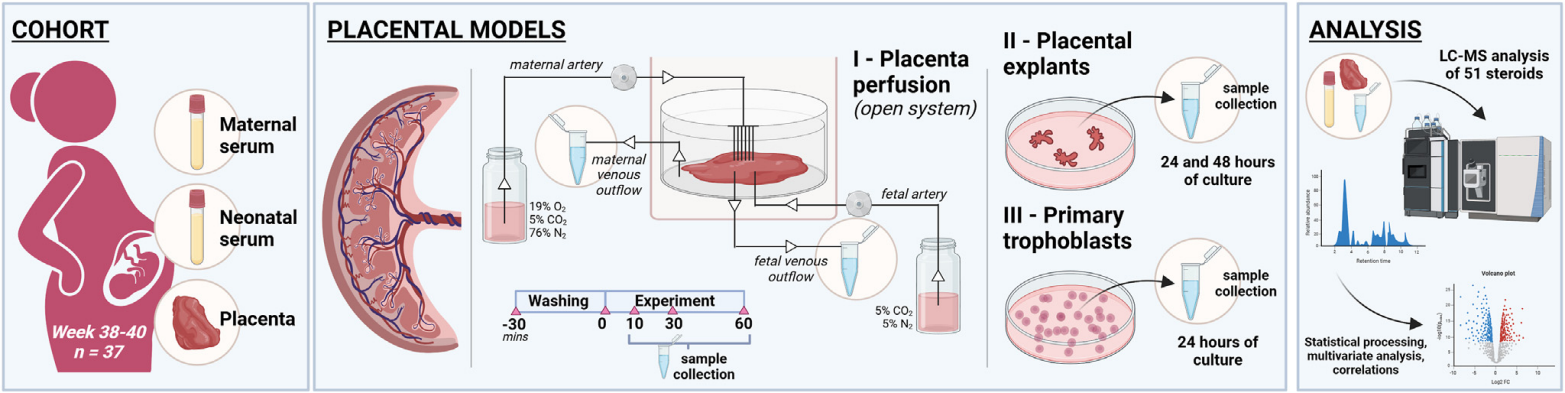
**Schematic representation of the study design.** Paired maternal serum, neonatal serum, and placental tissue samples were collected from 37 healthy pregnancies (gestational age at delivery: 38–40 weeks). To investigate the role of the placenta in the maternal-placental-fetal unit, we utilized advanced placental models, including (I) human placenta perfusion, (II) human placental explants, and (III) primary trophoblast cells isolated from term placentas. Steroid concentrations were measured in both the clinical samples and placental models using liquid chromatography-mass spectrometry (LC-MS). Statistical analyses, including multivariate approaches and correlation analyses, were employed to comprehensively interpret the data. Created in BioRender. Karahoda, R. (2025) https://BioRender.com/c24r796.

To address these gaps, we conducted a comprehensive study involving a cohort of 37 healthy human pregnancies. In this context, we analyzed the concentrations of both classic and novel steroids in paired maternal and neonatal serum, and placental samples. To further delineate the placental steroidogenic contributions and its differential secretion patterns, we utilized complementary *ex vivo* placental models, including human placenta perfusion, explants, and primary trophoblast cells (Fig. 1). These models provide distinct and complementary insights: placental perfusion preserves tissue architecture and circulation, allowing for a physiological assessment of steroid release to the maternal and fetal sides, as well as placental metabolism (37); placental explants retain structural complexity, enabling the study of local steroid synthesis and metabolism (38); and primary trophoblast cells offer a controlled environment to examine trophoblast-specific steroid release and mechanistic pathways (22). By integrating these approaches, our study provides a comprehensive evaluation of placental steroid metabolism from both tissue-level and cellular perspectives.

## Experimental Procedures

### Reagents

Methanol and acetonitrile (all LC–MS grade) were from Biosolve. Zinc sulfate heptahydrate and dichloromethane were obtained from Sigma-Aldrich; double charcoal stripped, delipidized human serum from Golden West Diagnostics; and formic acid and PBS tablets from Thermo Fisher Scientific. Steroid standards and internal standards were obtained either as certified reference solutions from Cerilliant or as powders from Steraloids.

### Study Approval

All pregnant women signed an informed consent form, and the sampling took place at the Division of Gynecology and Obstetrics of the Lindenhofgruppe. The study was conducted in accordance with the Declaration of Helsinki, and the protocol was approved by the Ethics Committee of the Canton of Bern (Basec Nr. 2016-00250).

### Sample Collection

Samples were collected from uncomplicated pregnancies (week 38–40) after elective caesarean section without prior labor symptoms upon patients’ request or due to breech presentation. The cohort comprises 37 paired maternal serum, neonatal serum, and placental tissue collected on the day of delivery. Additionally, for the perfusion, explant, and trophoblast experiments, 16 extra placentas were collected and processed. The demographic characteristics of study participants are summarized in supplemental Table S1.

### Human Placenta Perfusion

The single cotyledon placenta perfusion system was employed, as previously described with minor modifications (39, 40). Briefly, on the chorionic plate, a fetal artery and vein were cannulated to set up a vascular circuit. Perfusion was initiated using Dulbecco’s modified Eagle medium and Earle’s balanced salt solution containing 2 g/L of glucose (Sigma), 10 g/L Dextran FP40 (Serva), and 40 g/L bovine serum albumin (Sigma). Both the maternal and the fetal side were perfused with the same medium. Subsequently, a selected placental cotyledon and its surrounding tissue were transferred to a temperature-controlled chamber (37 °C), where perfusion of the fetal circulation was continued as an open system at a flow rate of 15 ml/hour/cannula. Depending on the cotyledon size, 5 to 25 cannulae were inserted at alternating depths of 1 and 2 cm into the intervillous space to establish the maternal circulation. Maternal flow rate was initiated at 0.5/ml/min/cannula (30 ml/hour/cannula) as an open system. The flow rate at the fetal side was 0.25 ml/min/cannula (15 ml/hour/cannula). The cylinder gas compositions for the maternal and fetal circuits were 19% O_2_/5% CO_2_/76% N_2_ and 5% CO_2_/95% N_2_, respectively. The maternal and fetal artery pressure was monitored by a Millar instrument (Millar instruments). The placental cotyledon perfusions that leaked secondary to lack of vascular integrity or that did not achieve a stable pressure were excluded from the study. After an initial 30 minutes washing phase, maternal and fetal venous perfusate samples (1 ml) were taken at 10, 30, 60 minutes (n = 3). Samples were collected in triplicate, centrifuged at 1100 g for 10 min at 4 °C, and the supernatant was stored at −80 °C until further analysis.

Several parameters were monitored throughout the perfusion experiment to assess the quality of the perfusion system (41). They include assessment of the volume of the fetal perfusate, pH, oxygen consumption, glucose consumption, lactate production, antipyrine, and creatinine transfer. The formulas used for the calculation of these parameters were previously described (39). The concentrations of antipyrine and creatinine added into the placenta perfusion medium were 80 and 150 μg/ml, respectively.

### Villous Term Placenta Explants

Pieces of cotyledons were dissected from human term placenta and the chorionic plate and decidua were removed. Villous tissue was dissected into explants and cleaned of large vessels and blood clots. The villous explants (approx. 100 mg explants/well) were rinsed with Mg/Ca free Hanks’ balanced salt solution and placed in Transwell inserts (Greiner Bio One, 3 μm pore size) for 6-well plates containing Explant Culture Medium for Placenta (Curio Biotech), enriched with 2.6 mM L-glutamine. Explants were incubated under 21% O_2_/5% CO_2_/72% N_2_ at 37 °C in a sterile incubator. After 24 and 48 h of incubation, the explant culture media (n = 9) were collected in triplicate, centrifuged at 1100*g* for 10 min at 4 °C, and the supernatants stored at −80 °C until further analysis. Villous explant viability and integrity was monitored by performing an established test based on 3-(4,5-dimethylthiazol-2-yl)-2,5-diphenyltetrazoliumbromide (Rose Scientific). In addition, histological assessment of formalin-fixed, paraffin-embedded explants was performed after H&E staining. Furthermore, to assure that our placental explant preparations are functionally and metabolically active during entire time frame of our experiments (48 h), we measured, among others, the release of human chorion gonadotropin into the medium.

### Primary Trophoblast Cells Isolated from Human Term Placenta and Cell Culture

Villous trophoblast cells were isolated from term placental tissue (n = 4) by enzymatic digestion and Percoll gradient separation, as previously described with minor modifications (42, 43, 44). The tissue was minced and digested three times with 0.25% trypsin (Sigma) and 300 IU/ml deoxyribonuclease I (Sigma) at 37 °C (30 min each). Cells were overlayed on a discontinuous Percoll (Sigma) density gradient. The purity of the isolated trophoblast cells was evaluated by analyzing the expression of cytokeratin-7 and vimentin as previously described (45, 46, 47). BeWo cells (clone b30) were donated by Dr Alan L. Schwartz (Washington University School of Medicine).

Thawed human epithelial trophoblast-like BeWo choriocarcinoma cells (passage 36–38) and freshly isolated primary human trophoblast cells were cultured at 37 °C with 5% CO_2_ atmosphere, at a density of 2 × 10^6^ cells/well in 6-well plates, using Dulbecco’s modified Eagle’s medium containing 4.5 g/L glucose (Gibco), enriched by 10% fetal bovine serum (charcoal-stripped) and 1 × antibiotic-antimitotic (Gibco). Culture media (2 ml) was collected at 24 h. Since primary trophoblast cells undergo spontaneous syncytialization over time, the 24-h time point was chosen to ensure analysis in their cytotrophoblast-like state. Samples were collected in triplicate, centrifuged at 1200*g* for 10 min at 4 °C, and the supernatant was stored at −80 °C until further analysis. For P4 spiking experiments, cells were treated with 1 μM progesterone, and the culture media was processed as described above.

### LC-MS Analysis

For the steroid analysis in explant medium, perfusates, and cell supernatants, 500 μl sample was spiked with 38 μl of a mixture of isotopically labeled standards in methanol (3.8 nM each). For maternal and neonatal serum, the sample volumes ranged from 80 to 500 μl; therefore, the sample volumes were corrected to 500 μl using PBS in water where needed and the dilution factors were incorporated to normalize the data. All calibrants (n = 12) and QC samples (high, middle, low) were prepared in double charcoal stripped, delipidized human serum as the matrix. Then, 250 μl of zinc sulfate (0.1 mol/L) and 500 μl of cold methanol (−20 °C) were added to the samples, the samples vortexed and centrifuged for 5 min at 8000 g (4 °C). Two hundred and fifty microliters of water was added to each sample, which was then purified using solid-phase extraction on an OasisPrime HLB 96-well plate using a positive pressure 96-well processor (both Waters). Samples were eluted using 250 μl pure acetonitrile which was subsequently dried under nitrogen, and samples were finally resuspended in 100 μl of 33% methanol in water.

For the analysis in placental tissue, 152.09 ± 28.52 mg tissue was spiked with 38 μl of a mixture of isotopically labeled standards and 1300 μl methanol added to each sample, the sample vortexed and centrifuged for 5 min at 3000 rpm (4 °C). The resulting supernatants were poured over into new Pyrex glass tubes and mixed with 3 ml methanol:dichloromethane (1:2) for 20 min using a general laboratory rotator. The resulting extract was centrifuged for 10 min at 3000 rpm (4 °C) and the organic layer dried under nitrogen. The placental extracts were finally dissolved in 200 μl 33% methanol. Steroid concentrations in placental tissue were normalized to the mg starting weight.

Separation of steroid metabolites (20 μl injection volume) was completed using a Vanquish UHPLC (from Thermo Fisher Scientific) with an Acquity UPLC HSS T3 column, 100 Å, 1.8 μm, 1 mm × 100 mm (from Waters) and detection using a Q Exactive Orbitrap Plus mass spectrometer (Thermo Fisher Scientific). Mobile phase A was water + 0.1% formic acid and B methanol + 0.1% formic acid. The separation gradient with a constant flow of 0.15 ml/min was as follows: 0 to 0.5 min 1% B, 0.5 to 1 min linear gradient to 1 to 46% B, 1 to 4 min 46%, 4 to 12 min linear gradient 46 to 73% B, 12–12.5-min linear gradient 73 to 99% B, 12.5 to 14.5 min 99% B, 14.5–15-min linear gradient to 1% B, 15 to 17 min 1% B. The mass spectrometer operated in both negative and positive ion modes using an electrospray ionization source and in both parallel reaction monitoring and full scan mode in a mass range of 200 to 500 m/z. All data were processed using TraceFinder 4.0 (Thermo Fisher Scientific). Please refer to Table 1 for the full list of steroids measured and their lower limit of quantifications and the previously published study for further details and method validation (9). The lower limit of quantification was determined at the lowest concentration for each steroid that can be quantitatively determined with acceptable precision and accuracy (9). Estrogens were not included in our multisteroid assay due to the requirement for derivatization to improve their detection limits. This process would have necessitated a more complex sample preparation procedure, which could have impacted the detection of other steroids and overall assay performance.

**Table 1:** List of all steroids measured in the study

Name	Abbreviation	LLOQ (nmol/L)
Pregnenolone	P5	0.771
17α-Hydroxypregnenolone	17OHP5	20.00
11-Deoxycortisol	S	0.088
Cortisol	F	0.378
Cortisone	E	0.177
11-Deoxycorticosterone	DOC	0.092
Corticosterone	Cort	0.705
Aldosterone	Aldo	0.085
Dehydroepiandrosterone sulfate	DHEA-S	6.252
Dehydroepiandrosterone	DHEA	0.846
Androstenediol	A5	20.00
Androstenedione	A4	0.107
Testosterone	T	0.105
Dihydrotestosterone	DHT	0.105
Androstanediol	3αDIOL	20.00
5α-Androstane-3,17-dione	5αDIONE	6.000
11β-Hydroxyandrostenedione	11OHA4	0.600
11β-Hydroxytestosterone	11OHT	0.150
11-Ketoandrostenedione	11KA4	0.600
11-Ketotestosterone	11KT	0.600
11β-Hydroxy-5α-androstane-3,17-dione	11OH5αDIONE	2.000
5α-Androstanetrione/11-keto-5α-androstane-3,17-dione	11K5αDIONE	2.000
11-Ketoandrostanolone/11-ketodihydrotestosterone	11KDHT	1.000
11β-Hydroxyandrosterone (11OHAST)	11OHAn	2.000
11-Ketoandrosterone (11KAST)	11KAn	2.000
Androsterone (AST)	An	0.420
Progesterone	P4	0.476
16α-Hydroxyprogesterone	16OHP4	0.600
6α-Hydroxyprogesterone	6αOHP4	0.600
6β-Hydroxyprogesterone	6βOHP4	1.000
11α-Hydroxyprogesterone	11αOHP4	1.500
11β-Hydroxyprogesterone	11βOHP4	0.100
11-Ketoprogesterone	11KP4	0.200
5α-Pregnanetrione/11-ketodihydroprogesterone	11KDHP4	1.000
17α-Hydroxyprogesterone	17OHP4	0.092
17α-Hydroxydihydroprogesterone (5α-pregnan-17α-ol-3,20-dione)	17OHDHP4	2.000
17α-Hydroxypregnanolone (5β-pregnan-3β,17α-diol-20-one)	17OHTHP	10.00
21-Deoxycortisol	21dF	0.088
17α20α-Dihydroxyprogesterone	17,20diOHP4	0.600
Pregnanetriol	Ptriol	15.00
Pregnanetriolone/11-ketopregnanetriol	11KPtriol	6.000
20α-Hydroxyprogesterone	20αOHP4	0.600
20β-Hydroxyprogesterone	20βOHP4	0.600
5α/β-Dihydroprogesterone	DHP4	6.000
Pregnanolone (3α,5β-THP)	THP	15.00
5α-Pregnanolone (3α,5α-THP; allopregnanolone)	5αTHP	10.00
5α/β-Pregnan-3β-ol-20-one (3β-hydroxy-5α-THP)	3βTHP	15.00
6α-Hydroxypregnanolone	6OHTHP	10.00
5α/β-Pregnan-3β, 20α-diol	20OH3βTHP	20.00
5α/β-Pregnan-3α, 20α-diol	20OHTHP	100.0

Steroids are grouped by steroid class (mineralo-/glucocorticoids, androgens, and progesterones), and within each class, they are ordered according to their biosynthetic pathway, maintaining a substrate-to-metabolite progression where possible, as illustrated in Figure 6.

∗For values in pg/ml, multiply the nmol/L value with the molecular weight of the analyte of interest.

LLOQ, lower limit of accurate quantification.

### Statistical Analysis

All statistical analyses were performed using GraphPad Prism version 10.1.2. Differences in the demographic characteristics of study participants were assessed using unpaired t-tests for continuous variables and Fisher’s exact test for categorical variables. Steroid concentrations below the lower limit of quantification were retained without replacement and were not included in statistical analyses. Multivariate analyses included principal component analysis (PCA) and Volcano plots to identify patterns and significant differences in steroid concentrations between maternal serum, neonatal serum, and placenta samples. The Volcano plots were based on the Wilcoxon matched-pairs signed rank test. Correlations between steroid levels and demographic variables were assessed using Spearman’s rank correlation coefficient. For time-dependent steroid release in the placenta perfusion model, a two-way ANOVA was used to evaluate differences over time. In the *ex vivo* explant model, time-dependent steroid release was analyzed using multiple paired t-tests. Similarly, the effect of P4 spiking on steroid release in primary trophoblast cells was assessed using multiple paired t-tests.

## Results

### Steroid Measurements in Maternal Serum, Neonatal Serum, and Placenta

**Fig. 2:**
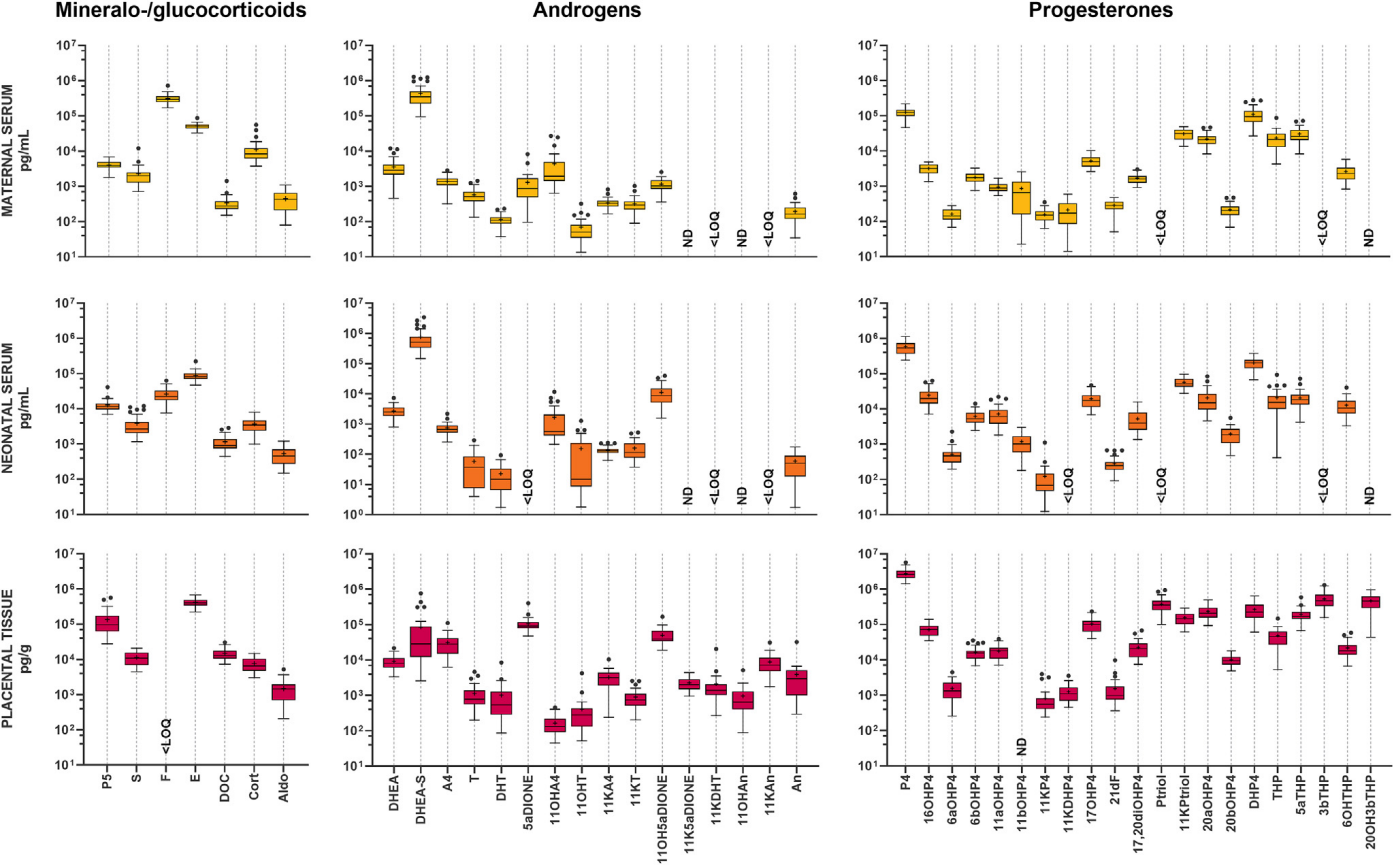
**Concentration of steroids in paired maternal serum, neonatal serum, and placenta.** Steroids are categorized into mineralo-/glucocorticoids, androgens, and progesterones. Data are presented as Tukey boxplots on a logarithmic scale. Steroids that were not detected (ND) or detected below the lower limit of accurate quantification (<LOQ) are denoted instead of a boxplot. Steroids that were only partially detected in some samples are detailed in supplemental Table S2. For abbreviations of steroid species, see text and Table 1.

In a cohort of 37 healthy pregnancies, paired maternal serum, neonatal serum, and placenta samples were analyzed for various steroids, which were categorized into mineralo-/glucocorticoids, androgens, and progesterones. The results indicate a diverse array of steroids detected across these biological compartments, providing insights into steroid dynamics during pregnancy (Fig. 2). Steroids that were only partially detected in some samples are detailed in supplemental Table S2.

In the mineralo-/glucocorticoid category, pregnenolone (P5), 11-deoxycortisol (S), cortisone (E), 11-deoxycorticosterone (DOC), corticosterone (Cort), and aldosterone (Aldo) were detected consistently across maternal serum, neonatal serum, and placenta. F showed variable detection, being present in maternal and neonatal serum but not in the placenta. Within the mineralo-/glucocorticoid category, F was predominant in maternal serum, whereas E was the dominant steroid in neonatal serum and placenta.

Among the androgens, dehydroepiandrosterone (DHEA), dehydroepiandrosterone sulfate (DHEA-S), A4, T, DHT, 11OHA4, 11β-hydroxytestosterone (11OHT), 11KA4, 11KT, 11β-hydroxy-5α-androstane-3,17-dione (11OH5αDIONE), and androsterone (An) were detected in all three compartments. Certain androgens exhibited variable detection across the compartments. Notably, 11β-hydroxyandrosterone (11OHAn) and 5α-androstanetrione were detected exclusively in the placenta. Additionally, 11-ketoandrosterone and 11-ketoandrostanolone (11KDHT) were detected below the limit of quantification in maternal and neonatal serum but were present in the placenta. 5α-androstane-3,17-dione (5αDIONE) was detected below the limit of quantification in neonatal serum. Androstenediol (A5) and androstanediol (3αDIOL) could not be detected in any of the biological compartments. DHEA-S was the androgen with the highest concentration in maternal and neonatal serum, whereas 5αDIONE dominated in the placenta. From the 11-oxy steroids, a domination of 11OHA4 in maternal and neonatal serum was observed, whereas in the placenta, 11KA4 was predominant.

In the category of progesterones, several steroids were detected in all three compartments, including, P4, 16α-hydroxyprogesterone (16OHP4), 6α-hydroxyprogesterone (6αOHP4), 6β-hydroxyprogesterone (6βOHP4), 11αOHP4, 11KP4, 17OHP4, 21dF, 17α20α-dihydroxyprogesterone (17,20diOHP4), pregnanetriolone (11-ketopregnanetriol; 11KPtriol), 20α-hydroxyprogesterone (20αOHP4), 20β-hydroxyprogesterone (20βOHP4), 5α/β-dihydroprogesterone (DHP4), pregnanolone (THP), 5α-pregnanolone (5αTHP), and 6α-hydroxypregnanolone (6OHTHP). 11βOHP4 was present in several samples of maternal and neonatal serum but was not detected in the placenta, whereas 5α/β-Pregnan-3β,20α-diol (20OH3βTHP) was exclusively detected in the placenta. Pregnanetriol (Ptriol) and 5α/β-pregnan-3β-ol-20-one (3βTHP) were detected below the lower limit of quantification in maternal and neonatal serum but were present in the placenta. 17-hydroxy-dihydroprogesterone (5α-pregnan-17α-ol-3,20-dione; 17OHDHP4), 17α-hydroxypregnanolone (5β-pregnan-3β,17α-diol-20-one; 17OHTHP), and 5α/β-Pregnan-3α, 20α-diol (20OHTHP) could not be detected in any of the biological compartments tested. Within the progesterone category, P4 was the dominant steroid in all biological compartments.

### Analysis of Steroid Profiles in Maternal and Neonatal Serum

**Fig. 3:**
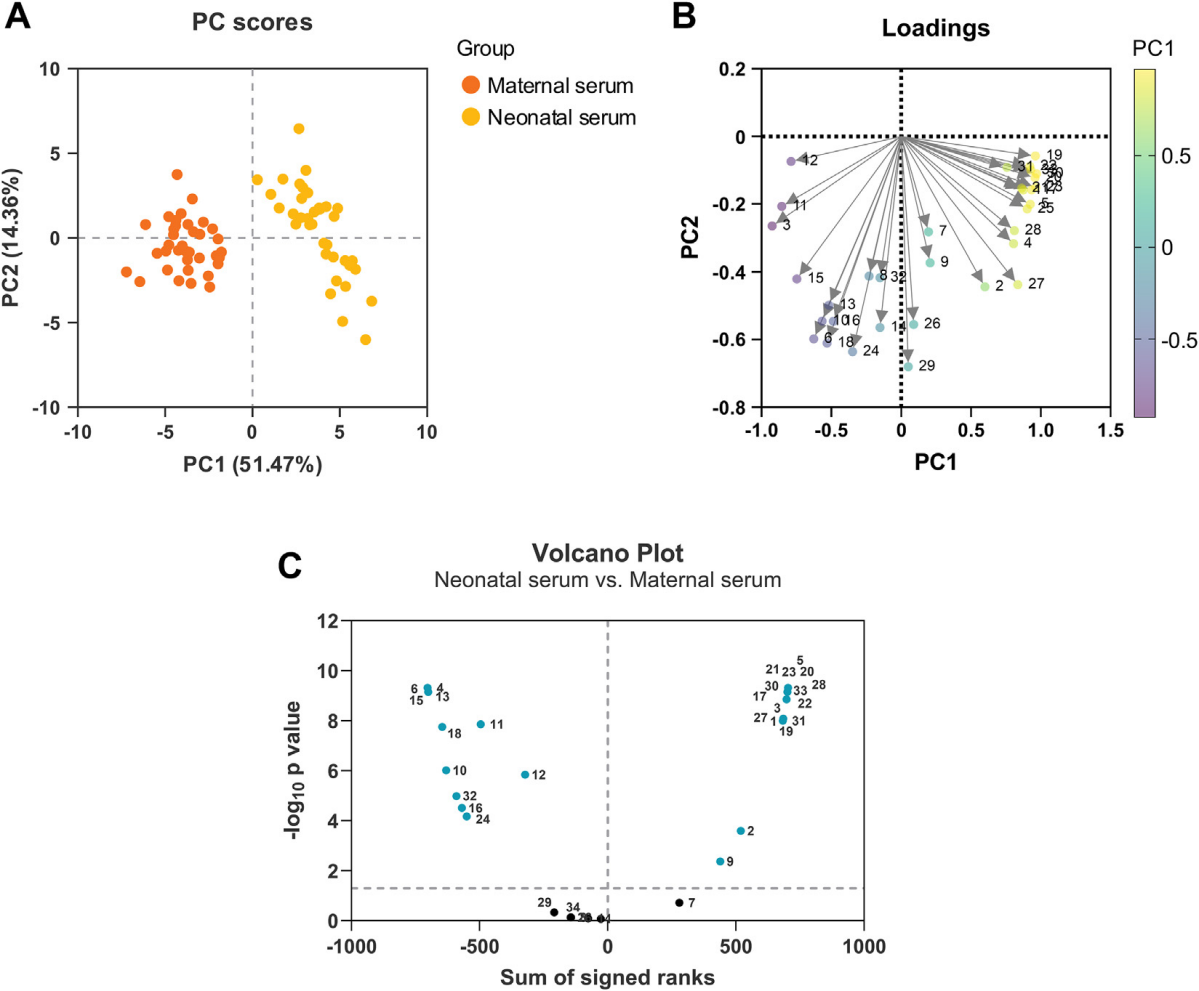
**Multivariate analysis of steroid concentrations in maternal and neonatal serum.***A*, principal component analysis (PCA) plot visualizing the clustering of maternal serum (*orange*) and neonatal serum (*yellow*) samples based on steroid concentrations. Positions of individual samples in the first and second components account for 51.47% and 14.36% of the variance in the data, respectively. *B*, loadings plot of the PCA highlighting the steroids with the most substantial positive contributions to the first principal component (PC1). *C*, volcano plot summarizing the intensity of differential concentrations between maternal and neonatal serum samples. The horizontal axis denotes the sum of signed ranks, and the vertical axis denotes -log_10_*p*-values. *Blue* dots represent steroids with statistically significant differences (*p* < 0.05), evaluated using Multiple Wilcoxon tests. *Number-coding: 1 – P5; 2 – S; 3 – E; 4 – F; 5 – DOC; 6 – Cort; 7 – Aldo; 8 – DHEA; 9 – DHEA–S; 10 – A4; 11 – T; 12 – DHT; 13 – 11OHA4; 14 – 11OHT; 15 – 11KA4; 16 – 11KT; 17 – 11OH5*α*DIONE; 18 – An; 19 – P4; 20 – 16OHP4; 21 – 6*α*OHP4; 22 – 6βOHP4; 23 – 11*α*OHP4; 24 – 11KP4; 25 – 17OHP4; 26 – 21dF; 27 – 17,20diOHP4; 28 – 11KPtriol; 29 – 20*α*OHP4; 30 – 20βOHP4; 31 – DHP4; 32 – 5*α*THP; 33 – 6OHTHP; 34 – THP.* For abbreviations of steroid species, see text and Table 1.

Next, we performed multivariate analysis to investigate the patterns of steroid concentrations in maternal and neonatal serum. PCA revealed distinct clustering of maternal serum separate from neonatal serum, indicating significant differences in steroid profiles between the two compartments. Maternal and neonatal serum were separated within the space spanned by the PCA model’s first two components, which explained 51.47% (PC1) and 14.36% (PC2) of the variance in the data (Fig. 3*A*).

Several steroids exhibited the highest positive loadings on the first PC (Fig. 3*B*). The steroids with the most substantial positive contributions to PC1 include 20βOHP4, 11αOHP4, P4, 16OHP4, 11OH5αDIONE, 6OHTHP, DOC, 6βOHP4, and 17OHP4. These findings highlight the critical role of progesterone derivatives and hydroxylated metabolites in defining the variance captured by PC1, suggesting their significant influence in distinguishing the samples based on their steroid profiles.

In addition, the volcano plot highlights steroids with significant differences in concentrations between maternal and neonatal serum (Fig. 3*C*). Steroids with high concentrations in neonatal serum include P5, P4, 16OHP4, 11αOHP4, 11KPtriol, 20βOHP4, and 6OHTHP. In contrast, steroids with high concentrations in maternal serum include F, 11KA4, Cort, and 11OHA4.

### Correlations of Steroid Profiles and Associations with Demographic Data

**Fig. 4:**
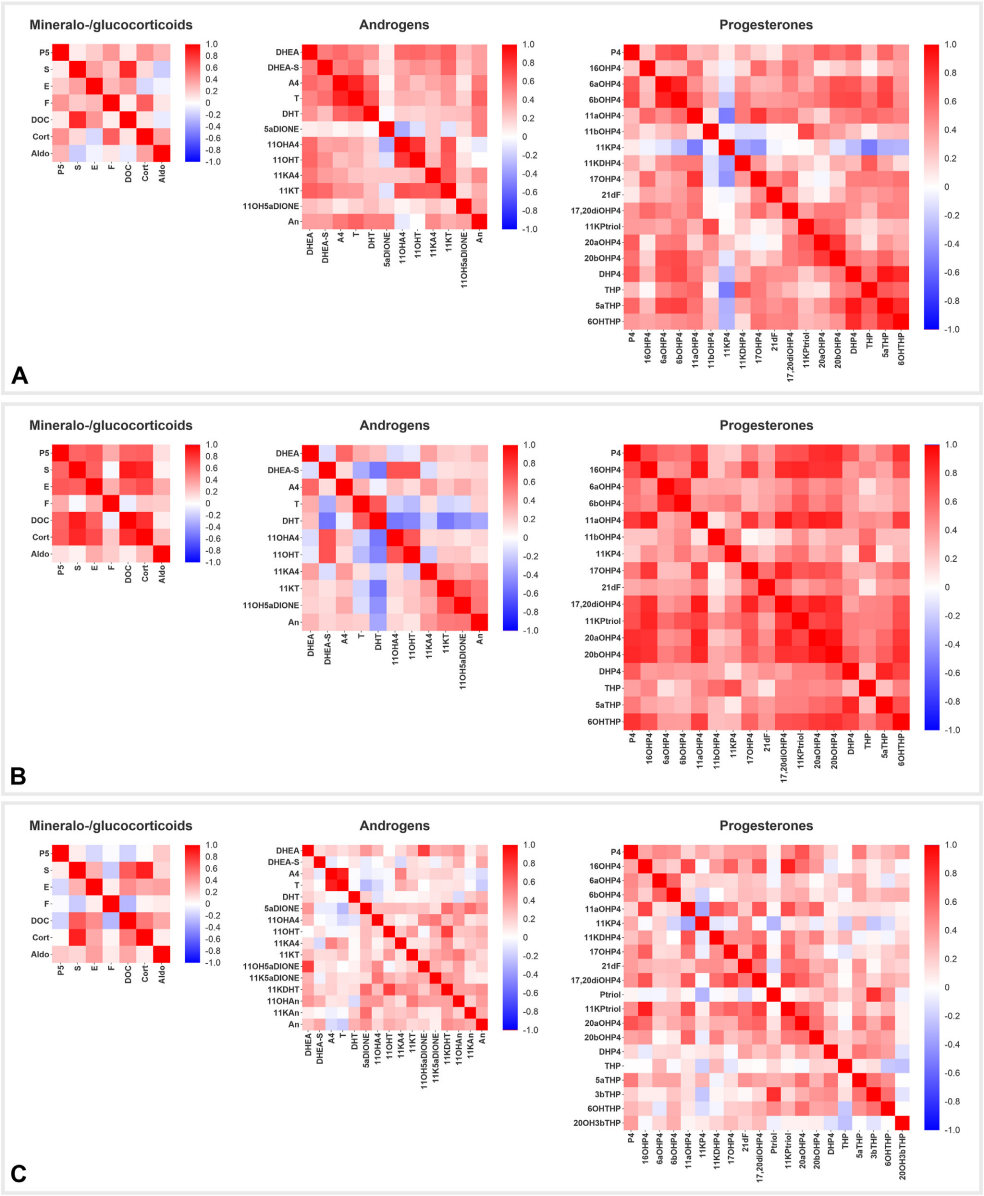
**Correlation heatmaps of steroid profiles.** Spearman’s rank correlation heatmap illustrating the relationships between mineralo-/glucocorticoids, androgens, and progesterones in maternal serum (*A*), neonatal serum (*B*), and placenta (*C*). The heatmaps are color-coded to indicate the strength and direction of correlations: *red* for positive correlations and *blue* for negative correlations. For abbreviations of steroid species, see text and Table 1.

Across all compartments (maternal serum, neonatal serum, and placenta), steroids within the same class (mineralo-/glucocorticoids, androgens, and progesterones) generally exhibit strong positive correlations, indicating coordinated regulation within these classes. However, there are notable differences in the strength and patterns of these correlations among the three compartments. In maternal serum, high and predominantly positive correlations are observed within the classes of progesterones and androgens (Fig. 4*A*). Neonatal serum shows the strongest positive correlations within the classes of mineralo-/glucocorticoids and progesterones (Fig. 4*B*). In the placenta, the correlations within steroid classes tend to be more moderate than maternal and neonatal serum (Fig. 4*C*).

In the class of mineralo-/glucocorticoids, a strong positive correlation cluster is observed in neonatal serum (Fig. 4*B*), comprising P5, S, and E. Moreover, there are strong positive correlations between Cort and DOC, as well as between Cort and DOC with P5, S, and E. In the maternal serum (Fig. 4*A*), a strong positive correlation cluster is seen among the classical androgens DHEA, DHEA-S, A4, T, and DHT. In neonatal serum (Fig. 4*B*), the strongest positive cluster is observed among the 11-oxy androgens 11KA4, 11KT, 11OH5αDIONE, and An. Notably, in both maternal and neonatal serum, 11OHA4 strongly positively correlates with 11OHT. In the placenta (Fig. 4*C*), the only strong positive correlation observed is between A4 and T. Lastly, progesterones show particularly strong positive correlations in both maternal and neonatal serum, with the highest correlations seen in neonatal serum. In maternal serum (Fig. 4*A*), a strong correlation cluster is observed among DHP4, THP, 5αTHP, and 6OHTHP. In neonatal serum (Fig. 4*B*), strong correlations are noted between 17,20diOHP4, 11KPtriol, 20αOHP4, and 20βOHP4, as well as their correlations with P4, 16OHP4, and 11αOHP4.

**Fig. 5:**
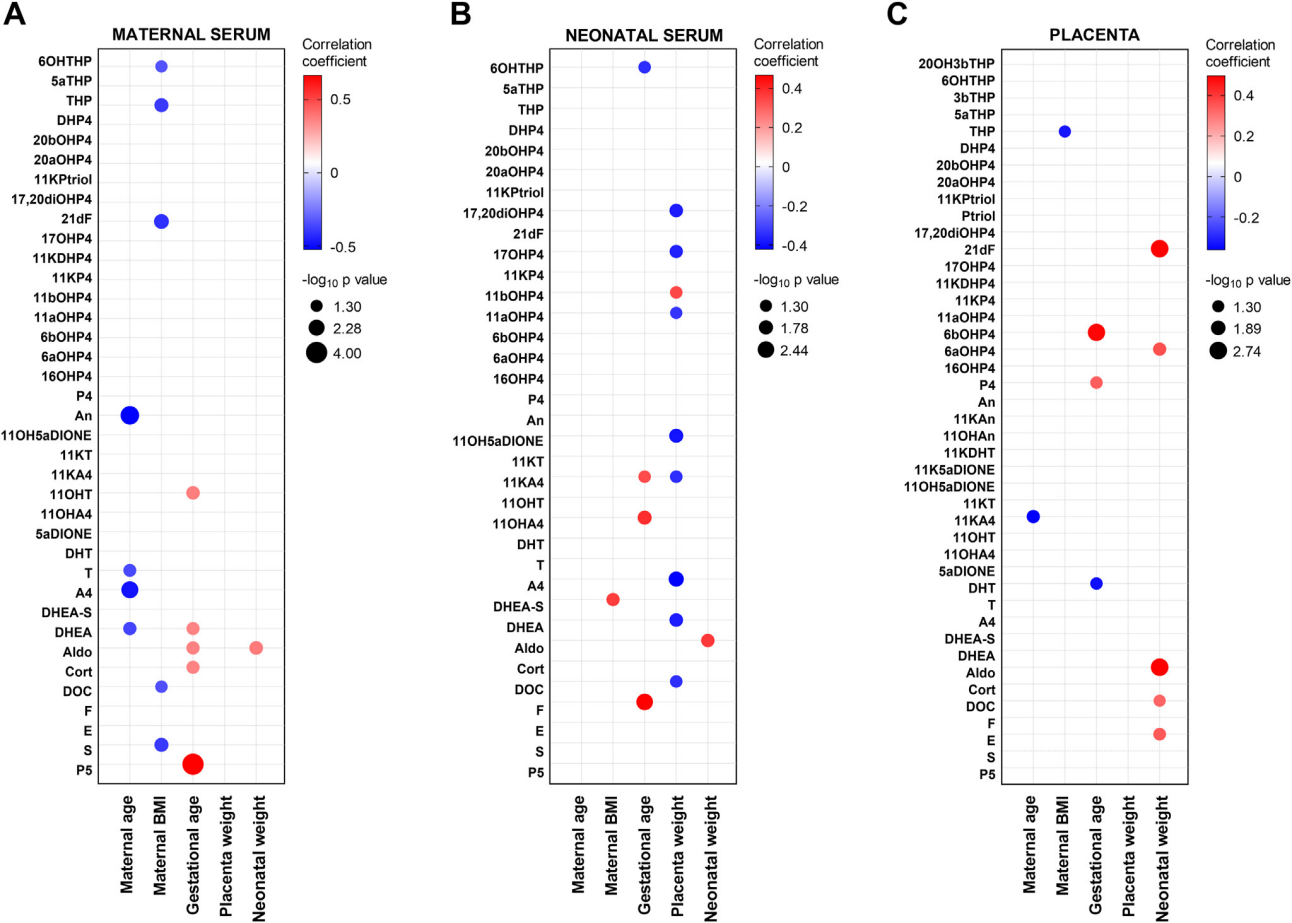
**Correlations between demographic patient data and individual steroid concentrations.** Bubble plot showing Spearman’s rank correlation coefficients between steroid concentrations in maternal serum (*A*), neonatal serum (*B*), and placenta (*C*), and demographic variables, including maternal age, maternal BMI, gestational age, placental weight, and neonatal weight. The correlation strength is indicated by the bubble color gradient, which is based on the correlation coefficient r, whereas the bubble radius represents the -log_10_*p*-values. Only significant correlations are indicated by the bubbles, which are restricted to metabolites with -log_10_*p*-values greater than 1.3. For abbreviations of steroid species, see Table 1.

We were also interested in examining how individual steroid concentrations correlate with available demographic data. Figure 5 presents the relationships between steroid levels and various demographic variables, including maternal age, maternal BMI, gestational age, placental weight, and neonatal weight. In terms of maternal age, we predominantly observed significant negative correlations with the concentrations of An, T, A4, and DHEA in maternal serum. Additionally, DOC and S in maternal serum were negatively correlated with maternal BMI (Fig. 5*A*).

Gestational age showed positive correlations with P5, Cort, Aldo, DHEA, and 11OHT in maternal serum (Fig. 5*A*). Similarly, gestational age was positively correlated with F, 11OHA4, and 11KA4 in neonatal serum (Fig. 5*B*). In the placenta, gestational age was positively correlated with P4 and 6βOHP4, while DHT showed a negative relationship with gestational age (Fig. 5*C*).

Placental weight showed significant negative correlations with several steroids in neonatal serum, specifically with DOC, DHEA, A4, 11KA4, 11OH5αDIONE, 11αOHP4, 17OHP4, and 17,20diOHP4 (Fig. 5*B*). Conversely, neonatal weight was positively correlated with placental concentrations of E, DOC, Aldo, 6αOHP4, and 21dF (Fig. 5*C*).

We also assessed whether the concentrations of steroids in the cohort exhibited sex-dependent changes. Overall, we observed minimal differences when comparing placentas from male and female newborns (supplemental Fig. S1). Specifically, the concentrations of DHT and T were significantly lower in the serum of female newborns than in male newborns (supplemental Fig. S1*B*). In the placenta, we observed lower levels of 3βTHP but higher levels of 11OHAn in female newborns than in males (supplemental Fig. S1*C*).

### Characterization of Product-To-Substrate Ratios in the Cohort

Next, we analyzed the product-to-substrate ratios to understand the activities of various steroid-metabolizing enzymes in maternal serum, neonatal serum, and placenta (supplemental Table S3). The findings highlight distinct enzymatic activities across these compartments, reflecting the unique metabolic environments. In terms of SRD5A activity, the placenta showed dominance in all processes, not only in the conversion of T to DHT but also in the metabolism of 11-keto steroids, including both androgens (11KA4 and 11KT) and progesterones (11KP4). This indicates a significant role of SRD5A in the placental metabolism of classic and novel steroids.

From the hydroxysteroid dehydrogenases, 17β-hydroxysteroid dehydrogenases showed high activity in the placenta, facilitating the reversible metabolism of 11KT to 11KA4. The enzyme 11βHSD2 also demonstrated high activity in the placenta, particularly in converting 11OHA4 to its keto derivative 11KA4 and to a lesser extent 11OHT to 11KT. However, because F was detected below the limit of quantification in the placenta, we could not compare it with the E/F ratios. In maternal serum, we observed predominant 11βHSD1 activity, whereas in neonatal serum, 11βHSD2 activity was more pronounced.

The enzyme CYP11B1 exhibited high activity in both maternal and neonatal serum, with minimal activity in the placenta. This enzyme is responsible for the hydroxylation of classical androgens A4 and T, as well as the hydroxylation of DOC and S to Cort and F, respectively. This distribution suggests a crucial role for CYP11B1 in the systemic circulation of steroids during pregnancy. Finally, the activities of AKR1C1, CYP17A1, and CYP21A2 were consistently low across all compartments, indicating that these enzymes presumably play a less significant role in the steroid metabolic processes examined in this study.

Overall, the data indicate distinct steroid metabolism patterns in maternal serum, neonatal serum, and placenta, with the placenta showing pronounced activity in converting hydroxylated steroids to keto derivatives, particularly for androgens, and demonstrating significant sulfotransferase activity.

### Steroid Release in *Ex Vivo/In Vitro* Placental Models

**Fig. 6:**
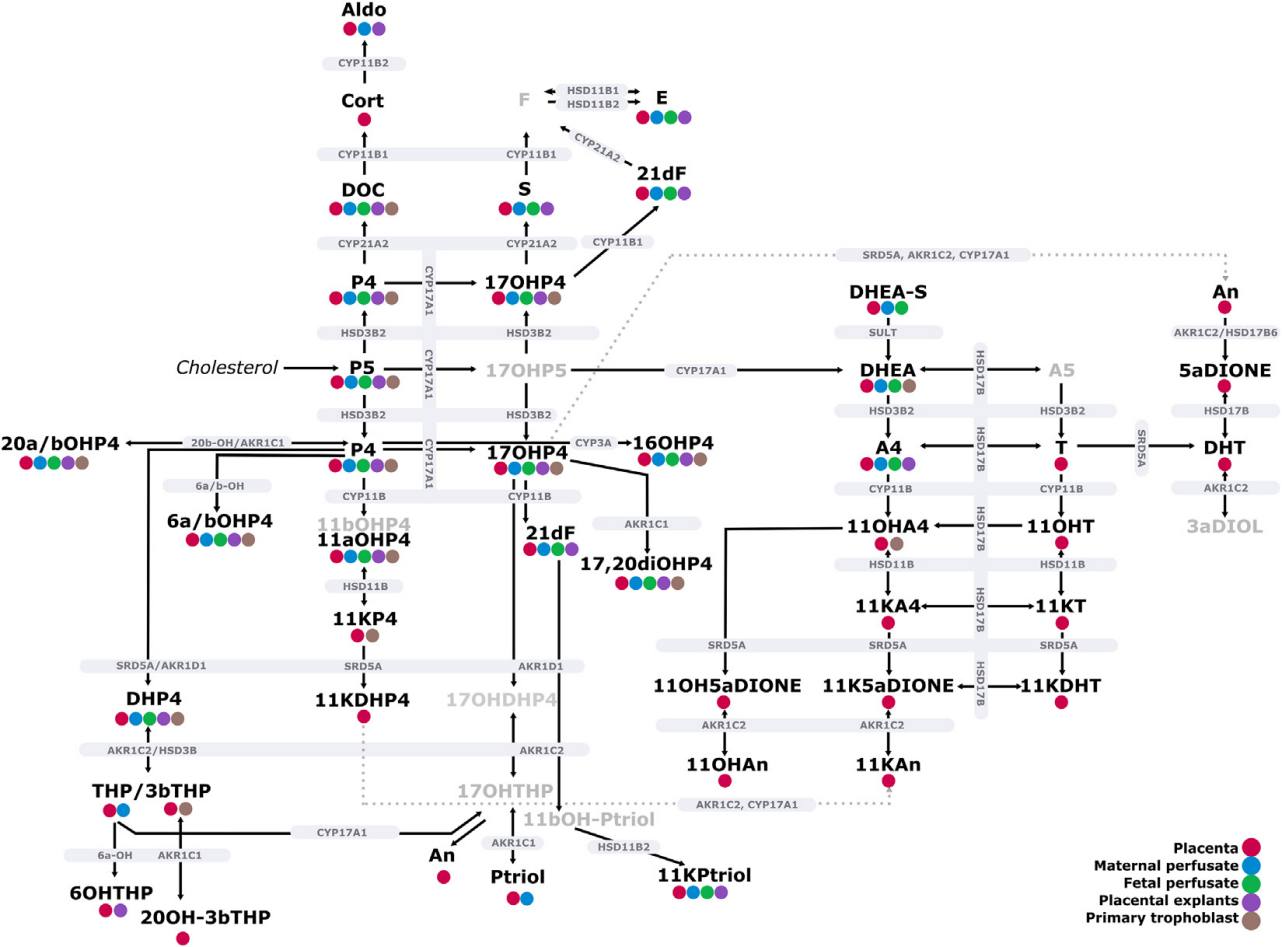
**Summary of steroid pathway across various placental systems.** The systems analyzed include human placenta tissue, human placenta perfusion (maternal and fetal perfusate), placental explants, and primary trophoblast cells isolated from human term placenta. The color coding represents the specific systems in which measurable concentrations of each steroid were detected. Steroids that were below the quantification limit or not detected are not highlighted by these colors. Steroids indicated in *light gray* represent absence across all systems. The backdoor pathway is represented by *dotted gray lines*. This visual representation highlights the dynamic steroidogenic activity within the placenta and its contribution in steroid synthesis and metabolism in the maternal-placental-fetal unit. While this figure depicts key steroidogenic enzymes involved in placental metabolism, evaluating their expression levels was beyond the scope of this study. For further details on placental enzyme expression, please refer to our previous study (22). For abbreviations of steroid species, see Table 1.

To evaluate placental steroid release, we utilized three complementary models: human placenta perfusion, placental explants, and primary trophoblast cells. Placental perfusion allowed for the measurement of steroid release on both the maternal and fetal sides, with samples collected at 10, 30, and 60 min (supplemental Figs. S2 and S3), while explant cultures provided insight into time-dependent steroid release over 24 and 48 h (supplemental Fig. S4). The results from these models, as summarized in Table 2 and Figure 6, reveal distinct patterns of steroid release across the different systems. P4 showed the highest release levels across all models, with particularly high concentrations observed in explants and maternal venous perfusate. P5 also exhibited substantial release, especially in the maternal and fetal venous perfusates, indicating its importance as the entry steroid metabolite into steroidogenesis. In the explant model, several progesterone derivatives such as 16OHP4, 6αOHP4, 6βOHP4, 11αOHP4, and 17OHP4 were released in significant amounts, with the highest levels detected at the 48-h time point (supplemental Fig. S4). This time-dependent increase highlights the sustained steroidogenic activity of placental tissue.

**Table 2:** Average steroid release in different placental models, including human placenta perfusion, explants, and human primary trophoblast cells

Steroid abbreviation	Maternal venous perfusate (pg/ml)	Fetal venous perfusate (pg/ml)	Placental explants (pg/ml)	Primary human trophoblast cells (pg/ml)
*P5*	499.67 ± 251.07	418.08 ± 58.03	413.63 ± 342.31	ND^a^
*S*	87.73 ± 7.03	23.38 ± 15.96	575.00 ± 304.51	ND
*F*	<LOQ	<LOQ	ND	ND
*E*	1557.43 ± 692.22	578.47 ± 139.85	11,737.79 ± 5366.96	ND
*DOC*	122.95 ± 24.07	49.73 ± 31.65	1856.53 ± 737.18	11.15 ± 5.85
*Cort*	<LOQ	<LOQ	<LOQ	ND
*Aldo*	2.38 ± 0.82	ND	58.84 ± 30.17	ND
*DHEA-S*	194.86 ± 59.17	38.08 ± 46.33	ND	<LOQ
*DHEA*	106.19 ± 35.85	102.38 ± 47.80	<LOQ	63.23 ± 53.59
*A4*	42.33 ± 21.38	18.20 ± 20.16	133.78 ± 63.48	<LOQ
*T*	<LOQ	<LOQ	<LOQ	ND
*11OHA4*	ND	ND	<LOQ	5.86 ± 1.40
*11KA4*	ND	ND	<LOQ	ND
*11OH5*α*DIONE*	<LOQ	<LOQ	<LOQ	ND
*11KAn*	<LOQ	<LOQ	ND	ND
*An*	ND	ND	<LOQ	ND
*P4*	73,769.43 ± 18,635.36	41,708.21 ± 7841.39	123,382.10 ± 46,553.73	48,876.76 ± 11,928.10
*16OHP4*	351.61 ± 44.68	66.64 ± 46.31	2599.93 ± 1356.06	586.43 ± 181.12
*6*α*OHP4*	20.08 ± 4.21	21.29 ± 8.83	1285.28 ± 502.44	157.81 ± 46.11
*6βOHP4*	192.66 ± 48.80	134.94 ± 17.41	13,018.03 ± 4446.77	752.74 ± 151.55
*11*α*OHP4*	119.04 ± 33.74	19.94 ± 18.65	1355.05 ± 592.37	33.96 ± 10.03
*11KP4*	ND	ND	ND	472.26 ± 155.64
*17OHP4*	257.68 ± 53.67	81.96 ± 37.26	1069.87 ± 674.99	19.91 ± 3.51
*21dF*	17.00 ± 5.82	4.55 ± 1.52	159.93 ± 94.93	ND
*17,20diOHP4*	281.41 ± 110.88	42.75 ± 24.41	639.14 ± 463.65	556.23 ± 45.71
*Ptriol*	1454.60 ± 141.67	<LOQ	<LOQ	ND^a^
*11KPtriol*	2880.00 ± 198.59	2888.08 ± 101.64	1990.07 ± 1065.97	ND^a^
*20*α*OHP4*	2957.56 ± 2042.51	601.66 ± 312.37	1244.88 ± 489.93	19,501.96 ± 12,312.06
*20βOHP4*	199.28 ± 70.03	91.27 ± 6.71	4869.37 ± 1888.20	739.83 ± 138.28
*DHP4*	8875.86 ± 4257.77	<LOQ	8869.03 ± 2189.91	3353.96 ± 482.09
*THP*	1920.38 ± 711.39	<LOQ	ND	ND^a^
*5*α*THP*	ND	ND	ND	953.65 ± 263.33
*3βTHP*	ND	ND	ND	13,565.42 ± 7896.66
*6OHTHP*	ND	ND	1642.77 ± 646,77	ND^a^

The results for the perfusion and explant model are shown as average at 60 min and 48 h, respectively. Values are presented as mean ± SD. For abbreviations of steroid species, see Table 1.

ND, not detected; <LOQ, detected below the lower limit of quantification.

a detected after P4 spiking.

DHEA and its sulfate form, DHEA-S, were prominently released in the perfusion models, particularly on the maternal side. DHEA levels were also notable in primary trophoblast cells, although DHEA-S was not detected in this model (supplemental Fig. S5). A4 was released in moderate amounts in the perfusion and explant models but was below the lower limit of quantification in trophoblast cells. Several steroids, such as 11OHA4, 11KA4, and 11OH5αDIONE, showed minimal or undetectable levels across all models. This indicates that these specific steroids are not major products of placental steroidogenesis or are produced in minor amounts below the quantification limit.

In the mineralo-/glucocorticoid group, E exhibited the highest release in explants, followed by maternal and fetal venous perfusates, whereas F was generally undetectable across all models. DOC showed the highest release in explants and was more predominant in maternal venous perfusate than in fetal venous perfusate, with very low levels in primary trophoblast cells. Aldo was detected in maternal venous perfusate and explants, but not in fetal venous perfusate or cells. S was present in all models, with the highest levels in explants, followed by maternal and fetal venous perfusates, and absent in cells.

When comparing steroid release between the maternal and fetal sides, several steroids were released on the maternal side in higher concentrations than on the fetal side, including P4, DHEA-S, 16OHP4, 11αOHP4, 17OHP4, 21dF, 17,20diOHP4, and DOC (Table 2). Additionally, certain steroids such as Ptriol, DHP4, THP, and Aldo were detected exclusively on the maternal side and not in the fetal venous perfusate, indicating a significant difference in the directionality of placental steroid release in the maternal and fetal side.

Interestingly, certain steroids were found to be undetectable in all experimental models, including A5, DHT, 5αDIONE, 3αDIOL, 11OHT, 11KT, 5α-androstanetrione, 11KDHT, 11OHAn, 17α-hydroxypregnenolone, 11βOHP4, 5α-pregnanetrione (11-ketodihydroprogesterone; 11KDHP4), 17OHDHP4, 17OHTHP, 20OHTHP, and 20OH3βTHP. This absence suggests that these steroids are either not synthesized by placental tissue or are present in very low concentrations.

### Steroid Release by Primary Trophoblast Cells

Next, we assessed steroid release from primary trophoblast cells under basal conditions and after P4 spiking to evaluate its impact on steroid production. Exogenous P4 significantly increased the release of multiple progesterones and led to the emergence of previously undetected steroids, including Ptriol, 11KPtriol, THP, 6OHTHP, and P5 (supplemental Fig. S5 and Table 2). This suggests that P4 acts both as a precursor and a regulatory stimulus, potentially enhancing enzymatic activity or shifting the metabolic flux to facilitate additional steroid biosynthesis.

Furthermore, we compared the steroid release profiles of primary trophoblast cells with those of immortalized BeWo cells, revealing distinct steroidogenic capacities. BeWo cells released higher concentrations of most steroids than primary trophoblast cells (supplemental Fig. S6). Additionally, P5, 17OHTHP, Ptriol, THP, and 6OHTHP were only released by BeWo cells under basal conditions and were not detected in primary trophoblast cells.

## Discussion

**Fig. 7:**
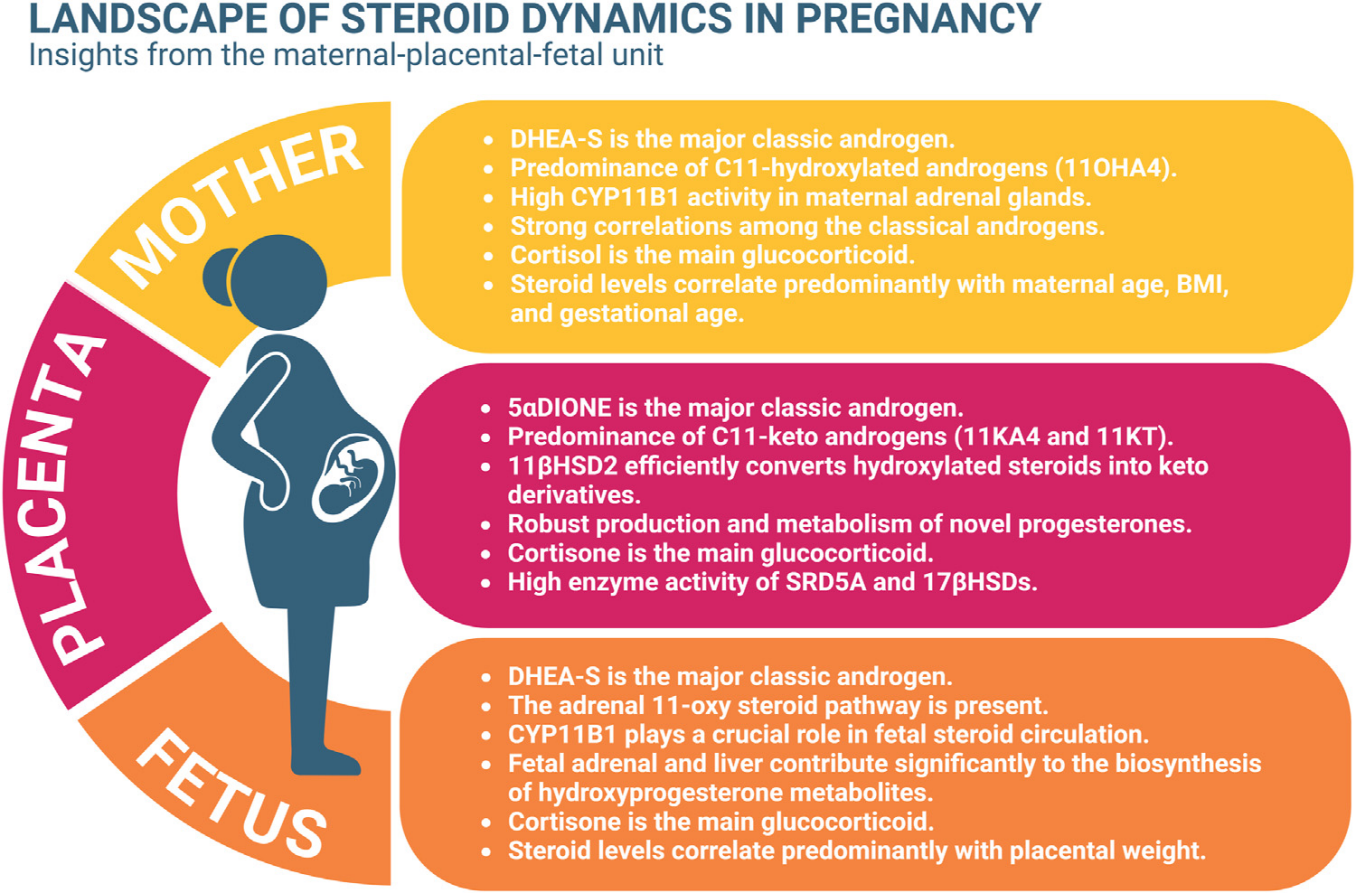
**Summary of steroid metabolism and trafficking across the maternal, placental, and fetal compartments during pregnancy.** The color scheme used to differentiate maternal serum, neonatal serum, and placenta in this figure is consistent with the color coding applied throughout the manuscript for clarity and uniformity in other figures. Created in BioRender. Karahoda, R. (2025) https://BioRender.com/f37c530.

This study provides a comprehensive analysis of steroid dynamics in pregnancy by measuring a broad range of steroids in paired maternal serum, neonatal serum, and placenta samples from 37 healthy pregnancies. We categorized steroids into mineralo-/glucocorticoids, androgens, and progesterones, analyzed maternal-neonatal differences, and explored correlations with demographic factors. Using placental perfusion, explants, and primary trophoblast cells, we further characterized placental steroid release. Our findings reveal the complex regulation of steroidogenesis in pregnancy and offer insights into the trafficking of steroid synthesis and metabolism within the maternal-placental-fetal unit (Fig. 7).

Steroid profiling revealed that 11OHA4 was the predominant C11-hydroxylated androgen in maternal and neonatal serum, while 11KA4 was the most abundant steroid in the placenta. These findings align with previous studies reporting a dominant role of 11-oxy androgens in maternal, neonatal, and placental compartments (10, 11, 12, 48). These patterns reflect the enzymatic interplay between the maternal and fetal compartments. The high circulating levels of A4 and T suggest that these classical androgens serve as substrates for adrenal CYP11B1, generating hydroxylated steroids (16, 17), which are further metabolized by placental 11βHSD2 into their keto derivatives (20). In our study, the elevated 11OHA4/A4 ratio in maternal serum suggests predominant CYP11B1 activity in the maternal adrenal glands. However, the strong correlation we observed between classical androgens in maternal serum and 11-oxy steroids in neonatal serum indicates active fetal steroidogenesis. Given that the fetal testes produce classical androgens only at specific developmental stages and the fetal ovary remains inactive throughout pregnancy (49, 50), the fetal and maternal adrenal glands likely serve as the primary sources of 11-oxy androgens. Supporting this, our placental models did not show the release of C11-hydroxylated steroids or their keto derivatives, reinforcing that the placenta metabolizes rather than synthesizes these androgens. This dual maternal and fetal contribution underscores the complexity of steroidogenesis in the maternal-placental-fetal unit and highlights the need for further research into its regulatory mechanisms.

The interplay between classical and 11-oxy steroids within the maternal-placental-fetal unit emphasizes the broader role of 11βHSD2 beyond glucocorticoid regulation. Traditionally recognized for protecting the fetus from excess maternal cortisol (27), its function in converting 11OHA4 to 11KA4 and 11OHT to 11KT, potent androgens comparable to T and DHT, has been largely overlooked (28, 29). Our study confirms the predominance of keto-derivatives (11KA4 and 11KT) over hydroxylated forms (11OHA4 and 11OHT) in the placenta, as also observed by Yoshida *et al.* (10), with a significant 11KA4/11OHA4 ratio indicating high placental 11βHSD2 activity. Beyond 11-oxy androgens, 11βHSD2 also contributes to 11-oxy progesterone metabolism, converting 11βOHP4 to 11KP4. However, in our study, the predominance of 11αOHP4 over 11KP4, along with the absence of 11βOHP4, suggests that 11βHSD2 may have limited activity toward 11-oxy progesterones. This is further supported by the substantial 11αOHP4 release observed in placental models, particularly on the maternal side in perfusion studies. Given this, CYP11B1 emerges as a potential contributor to 11αOHP4 production, despite its minimal placental activity (indexed by the product-to-substrate ratio). However, the reported presence of CYP11B1 transcripts in the placenta (22) suggests that with high P4 concentrations, even low enzymatic activity may be sufficient to generate 11αOHP4. Alternatively, other C11-hydroxylating CYP enzymes in the placenta may play a role, warranting further investigation.

Substantial amounts of 16OHP4 were detected in maternal serum, neonatal serum, and placenta, suggesting active 16-hydroxylase activity. Its concentrations were comparable to 17OHP4, consistent with previous reports in neonatal plasma and placental blood (12, 51). While fetal adrenal CYP17A1 and hepatic CYP3A7 have been identified as primary sources of 16OHP4 (52), its high levels across all compartments, including maternal serum, suggest additional contributions. All placental models released significant amounts of 16OHP4, with higher perfusate levels on the maternal side, indicating a potential placental role in its regulation. Additionally, the 16OHP4/P4 ratio was highest in neonatal serum, while maternal serum and placenta had similar ratios, reinforcing the dominance of fetal production but also suggesting placental and maternal contributions. The detection of *CYP3A* mRNA in human placenta (53, 54) supports the likelihood of intraplacental P4 C16-hydroxylation, suggesting that the placenta may actively contribute to 16OHP4 production. Despite this, the physiological role of 16OHP4 remains unclear, warranting further investigation to determine its functional significance within the maternal-placental-fetal unit (55).

Beyond 16OHP4, other hydroxylated progesterones, particularly 11-oxy progesterones, may play a significant role in steroid metabolism within the maternal-placental-fetal unit. While research on these compounds remains limited, they have been detected in neonates (12, 31) and may contribute to the pool of active androgens through metabolic conversion. Notably, 21dF and 11OHP4 can be converted into 11-oxy androgen precursors of 11KDHT (33, 34), potentially influencing androgen availability. Additionally, these 11-oxy progesterones have recently been linked to androgen and progesterone receptor activity (56), further highlighting their functional significance. In addition, 17OHP4 contributes to androgen biosynthesis via the backdoor pathway, bypassing conventional intermediates to produce DHT (35). In our study, high levels of 17OHP4, 21dF, 17,20diOHP4, Ptriol, and 11KPtriol were detected in the placenta and all placental models. Placental perfusion studies revealed a predominant release of these metabolites to the maternal side, indicating that the placenta actively metabolizes these compounds rather than simply serving as a transfer site. Further supporting this, P4 spiking in primary trophoblast cells significantly increased the release of multiple progesterones, including previously undetected compounds. This suggests that exogenous P4 not only serves as a precursor but may also modulate enzymatic activity or redirect metabolic pathways, leading to the synthesis of additional steroid-derived compounds. These findings highlight the active role of the placenta in steroid metabolism, particularly in the regulation of 11-oxy and backdoor androgen pathways, requiring further investigation into their physiological significance.

In the cohort, we were also interested in investigating the correlations between specific steroid concentrations and the demographic data available. Our analysis revealed significant associations between various steroids and demographic factors such as maternal age, maternal BMI, gestational age, placental weight, and neonatal weight. While we expected clear sexual dimorphism in placental steroid concentrations, the differences were minimal. Only a few steroids, including 3βTHP and 11OHAn, showed sex-dependent differences, while most remained comparable between male and female placentas. This aligns with previous findings (10), suggesting that pronounced placental sex-specific differences may occur earlier, during the critical period of sex determination (6).

Our study has several notable strengths. The use of paired samples enabled a comprehensive evaluation of both systemic circulation and local placental metabolism, providing an integrated perspective on steroid dynamics during pregnancy. Additionally, our advanced analytical method quantified over 50 steroids (9), far exceeding traditional clinical panels that typically measure fewer than 15, allowing for a more complete characterization of steroid metabolism. Finally, we employed three complementary human placental models, each providing distinct insights into placental function and steroid regulation (Fig. 6).

However, our study has a few limitations that should be considered. From a technical/methodological perspective, estrogens were not included in our multisteroid assay, preventing assessment of their role in the maternal-placental-fetal unit. Given the role of CYP19A1 in aromatization, measuring estrogen and 11-oxy estrogens (57) would enhance the understanding of placental steroid metabolism. Additionally, the lack of internal deuterated reference standards may impact absolute quantification, though our approach still allows for robust comparative analysis. Moreover, exogenous precursors were not supplemented in the culture media, except in the P4 spiking experiment, which may have limited the detection of certain metabolites. Future studies should incorporate specific precursors, estrogen profiling, and internal standards to enhance accuracy and fully characterize placental steroid metabolism.

From a biological perspective, our study focused on term placentas and blood samples, despite known gestational variations in steroidogenesis (22). Since 11-oxy androgens increase in maternal circulation across pregnancy (11), studying earlier developmental stages is crucial for capturing the dynamic regulation of steroid metabolism throughout gestation. Additionally, our placental models primarily assessed the villous cytotrophoblast phenotype, while extravillous trophoblasts (EVTs) were not considered. Transcriptomic data from early gestation indicate that EVTs express key steroid-metabolizing enzymes, including *SRD5A1*, *HSD11B2*, *HSD17B1*, and *CYP19A1*, with higher *SRD5A1* expression in EVTs than in villous cytotrophoblasts (58). Moreover, the same study reported active secretion of progesterone by EVTs, challenging the conventional view that syncytiotrophoblasts are the primary placental source of this hormone (58). Given that EVTs interact directly with the maternal circulation, they may play an important role in local steroid metabolism, decidual adaptation, and immune modulation during early pregnancy. Future research should further investigate EVT steroidogenesis in early gestation, utilizing metabolomic approaches to elucidate its role in pregnancy maintenance and maternal-fetal immune interactions.

In summary, we observed high levels of classic and novel progesterones in the placenta and all placental models, with a predominant release of these metabolites to the maternal side in the placenta perfusion model. This supports the idea that the placenta possesses a functional enzymatic machinery capable of producing and metabolizing these novel progesterones. Additionally, we demonstrated that 11βHSD2 activity extends beyond cortisol metabolism to include androgens, such as 11OHA4 and 11OHT, highlighting its broader role in steroid metabolism within the maternal-placental-fetal unit. The emerging role of 11βHSD2 beyond cortisol deactivation is particularly intriguing, as E/F ratios have been associated with pregnancy complications such as preeclampsia and fetal growth restriction (59, 60, 61). Given that 11OHA4/11KA4 ratios align with E/F ratios (11) and 11OHA4 has comparable binding affinities for 11βHSD2 as F (36), evaluating 11-oxy steroids in pregnancy complications could provide novel insights into their pathophysiology. Future studies should assess these steroids in compromised pregnancies to explore their potential as biomarkers for pregnancy-related disorders.

## Data availability

All data supporting the findings of this study are available within the article and its supplementary materials.

## Supplemental data

This article contains supplemental data.

## Conflicts of interest

The authors declare that they have no conflicts of interest with the contents of this article.
