# Supplementary material for: Landscape of Steroid Dynamics in Pregnancy: Insights From the Maternal-Placental-Fetal Unit and Placental Models
Source: Mol Cell Proteomics. 2025 Apr 23;24(6):100976. doi: 10.1016/j.mcpro.2025.100976 (PMC12289528; doi:10.1016/j.mcpro.2025.100976)
Supplement: Supporting_Information [file mmc1.docx]

**SUPPLEMENTARY DATA**

**Supplementary Table 1.** Demographic characteristics of study participants. Data are presented as mean ± standard deviation or as ratios where applicable.

| **Parameter** | **Paired serum and placenta (n=37)** | **Placenta for ex vivo/in vitro studies (n=16)** | **p value** |
| --- | --- | --- | --- |
| Gestational age (weeks) | 38.74 ± 1.18 | 38.46 ± 0.72 | 0.3949 |
| Maternal BMI at delivery (kg/m^2^) | 29.52 ± 4.40 | 31.08 ± 5.80 | 0.3166 |
| Maternal age (years) | 33.76 ± 4.23 | 35.00 ± 4.15 | 0.3275 |
| Smoking (Y:N) | 2:35 | 2:14 | 0.5754 |
| Neonatal weight (kg) | 3.41 ± 0.40 | 3.52 ± 0.37 | 0.3164 |
| Neonatal sex (M:F) | 18:17 | 11:5 | 0.3623 |
| Placental weight (kg) | 0.64 ± 0.11 | 0.67 ± 0.10 | 0.3157 |
| Delivery (CS:S) | 35:2 | 16:0 | >0.9999 |

Statistical analyses were performed using unpaired t-tests for continuous variables and Fisher’s exact test for categorical variables. *Abbreviations: CS - cesarean section, F - female, M - male, N - no, S - spontaneous, Y - yes.*

**Supplementary Table 2.** Steroids that were partially detected across maternal serum, fetal serum, and placenta. A total of 37 samples were analyzed - the "Samples <LOQ" column indicates how many samples had steroid concentrations below LOQ, while the remaining samples had detectable levels.

| **System** | **Steroid** | **Samples <LOQ (out of 37)** |
| --- | --- | --- |
| Maternal Serum | 11KDHP4 | 14 |
| Maternal Serum | 11βOHP4 | 13 |
| Maternal Serum | 5αDIONE | 5 |
| Maternal Serum | THP | 8 |
| Fetal Serum | DHT | 15 |
| Fetal Serum | 11βOHP4 | 15 |
| Fetal Serum | T | 10 |
| Fetal Serum | 11OHT | 4 |
| Fetal Serum | THP | 3 |
| Fetal Serum | An | 3 |
| Fetal Serum | 11KT | 1 |
| Placenta | THP | 7 |
| Placenta | 11KA4 | 1 |
| Placenta | An | 1 |
| Placenta | 11OHAn | 1 |

**Supplementary Table 3.** Apparent activities of steroid-metabolizing enzymes calculated as product-to-substrate ratio. In cases where one of the metabolites (either the product or the substrate) was not detected, the ratio could not be calculated. Data are presented as mean with standard error of the mean (SEM); high and low values are highlighted using a heatmap format. Abbreviations of steroid species see Table 2 (manuscript file).

| **Product-to-substrate ratio** | **Maternal serum** | | | **Neonatal serum** | | | **Placenta** | | |
| --- | --- | --- | --- | --- | --- | --- | --- | --- | --- |
|  | **mean** | **SEM** | **n** | **mean** | **SEM** | **n** | **mean** | **SEM** | **n** |
| 17βHSD (T / A4) | 0.414 | 0.017 | 37 | 0.079 | 0.022 | 27 | 0.036 | 0.003 | 37 |
| 17βHSD (11KT / 11A4) | 0.948 | 0.052 | 37 | 1.185 | 0.157 | 36 | 0.478 | 0.119 | 36 |
| 17βHSD (11KA4 / 11KT) | 1.188 | 0.075 | 37 | 1.284 | 0.133 | 36 | 5.201 | 0.832 | 36 |
| SRD5A (DHT / T) | 0.221 | 0.013 | 37 | 0.664 | 0.25 | 19 | 1.429 | 0.314 | 37 |
| SRD5A (11K5αDIONE / 11KA4) | - | - | - | - | - | - | 1.251 | 0.419 | 36 |
| SRD5A (11KDHT / 11KT) | - | - | - | - | - | - | 2.632 | 0.428 | 37 |
| SRD5A (11KDHP4 / 11KP4) | 1.737 | 0.271 | 23 | - | - | - | 2.472 | 0.365 | 37 |
| SRD5A/AKR1D1 (DHP4 / P4) | 0.864 | 0.055 | 37 | 0.382 | 0.025 | 37 | 0.103 | 0.009 | 37 |
| 3βHSD (P4 / P5) | 33.144 | 2.093 | 37 | 46.651 | 2.47 | 37 | 30.583 | 3.307 | 37 |
| 3βHSD (A4 / DHEA) | 0.476 | 0.036 | 37 | 0.305 | 0.024 | 37 | 3.838 | 0.417 | 37 |
| Sulfotransferase (DHEA / DHEAS) | 0.01 | 0.001 | 37 | 0.007 | 0.001 | 37 | 0.474 | 0.09 | 37 |
| CYP11B1 (11OHA4 / A4) | 3.132 | 0.55 | 37 | 2.339 | 0.501 | 37 | 0.008 | 0.001 | 37 |
| CYP11B1 (11OHT / T) | 0.14 | 0.019 | 37 | 6.991 | 2.97 | 25 | 0.549 | 0.134 | 37 |
| CYP11B1 (F / S) | 180.946 | 16.244 | 37 | 9.473 | 1.129 | 37 | - | - | - |
| CYP11B1 (CORT / DOC) | 42.106 | 8.206 | 37 | 3.395 | 0.165 | 37 | 0.547 | 0.048 | 37 |
| CYP11B (11αOH) (11αOHP4 / P4) | 0.008 | 0.001 | 37 | 0.012 | 0.001 | 37 | 0.007 | 0.001 | 37 |
| CYP11B (11βOH) (11βOHP4 / P4) | 0.007 | 0.001 | 24 | 0.002 | 0 | 22 | - | - | - |
| 11βHSD2 (11KA4 / 11OHA4) | 0.167 | 0.016 | 37 | 0.234 | 0.026 | 37 | 27.557 | 4.337 | 36 |
| 11βHSD2 (11KT / 11OHT) | 5.95 | 0.574 | 37 | 10.476 | 2.031 | 32 | 3.787 | 0.434 | 37 |
| 11βHSD1 (F / E) | 6.284 | 0.417 | 37 | 0.307 | 0.026 | 37 | - | - | - |
| 11βHSD2 (E / F) | 0.176 | 0.008 | 37 | 3.894 | 0.254 | 37 | - | - | - |
| 11βHSD2 (11KP4 / 11βOHP4) | 1.056 | 0.448 | 24 | 0.21 | 0.065 | 22 | - | - | - |
| AKR1C2 (11KAn / 11K5αDIONE) | - | - | - | - | - | - | 4.359 | 0.443 | 37 |
| AKR1C2/3βHSD (3βTHP / DHP4) | - | - | - | - | - | - | 2.43 | 0.289 | 37 |
| AKR1C1 (20α-HSD) (20αOHP4 / P4) | 0.18 | 0.009 | 37 | 0.033 | 0.003 | 37 | 0.086 | 0.004 | 37 |
| AKR1C1 (20β-HSD) (20βOHP4 / P4) | 0.002 | 0 | 37 | 0.003 | 0 | 37 | 0.004 | 0 | 37 |
| AKR1C1 (20OH-3βTHP / 3βTHP) | - | - | - | - | - | - | 1.163 | 0.161 | 37 |
| CYP21A2 (S / 17OHP4) | 0.419 | 0.031 | 37 | 0.194 | 0.009 | 37 | 0.118 | 0.005 | 37 |
| CYP21A2 (DOC / P4) | 0.003 | 0 | 37 | 0.002 | 0 | 37 | 0.006 | 0 | 37 |
| CYP17A1 (17OHP4 / P4) | 0.046 | 0.003 | 37 | 0.035 | 0.002 | 37 | 0.04 | 0.004 | 37 |
| CYP17A1/hep. CYPs (16OHP4 / P4) | 0.028 | 0.002 | 37 | 0.043 | 0.003 | 37 | 0.027 | 0.002 | 37 |
| 6α-OH (6αOHP4 / P4) | 0.001 | 0 | 37 | 0.001 | 0 | 37 | 0.001 | 0 | 37 |
| 6β-OH (6βOHP4 / P4) | 0.015 | 0.001 | 37 | 0.012 | 0.001 | 37 | 0.006 | 0 | 37 |
| 6α-OH (6OHTHP / 3βTHP) | - | - | - | - | - | - | 0.045 | 0.004 | 37 |


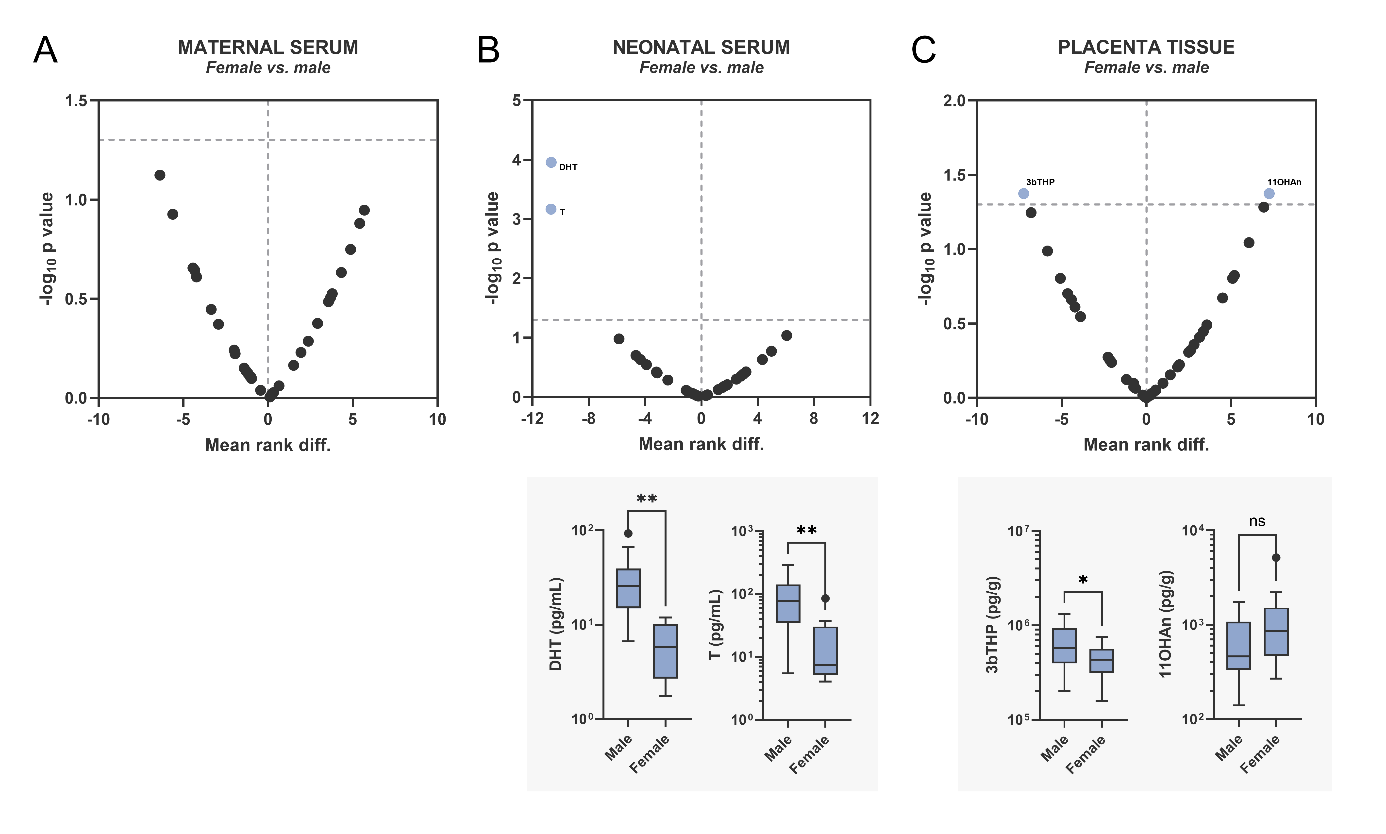


**Supplementary Figure 1.** Sex-dependent patterns in steroid concentrations. Volcano plots and boxplots illustrating sex-dependent patterns in steroid concentrations across maternal serum (A), neonatal serum (B), and placenta (C). The horizontal axis in volcano plots denote the sum of signed ranks, and the vertical axis -log_10_ p-values. Blue dots represent steroids with statistically significant differences (p < 0,05*; p < 0,01**), evaluated using multiple Mann-Whitney tests. The boxplots presented on a logarithmic scale show the distribution of steroid levels, highlighting only the steroids in which significant differences were observed.


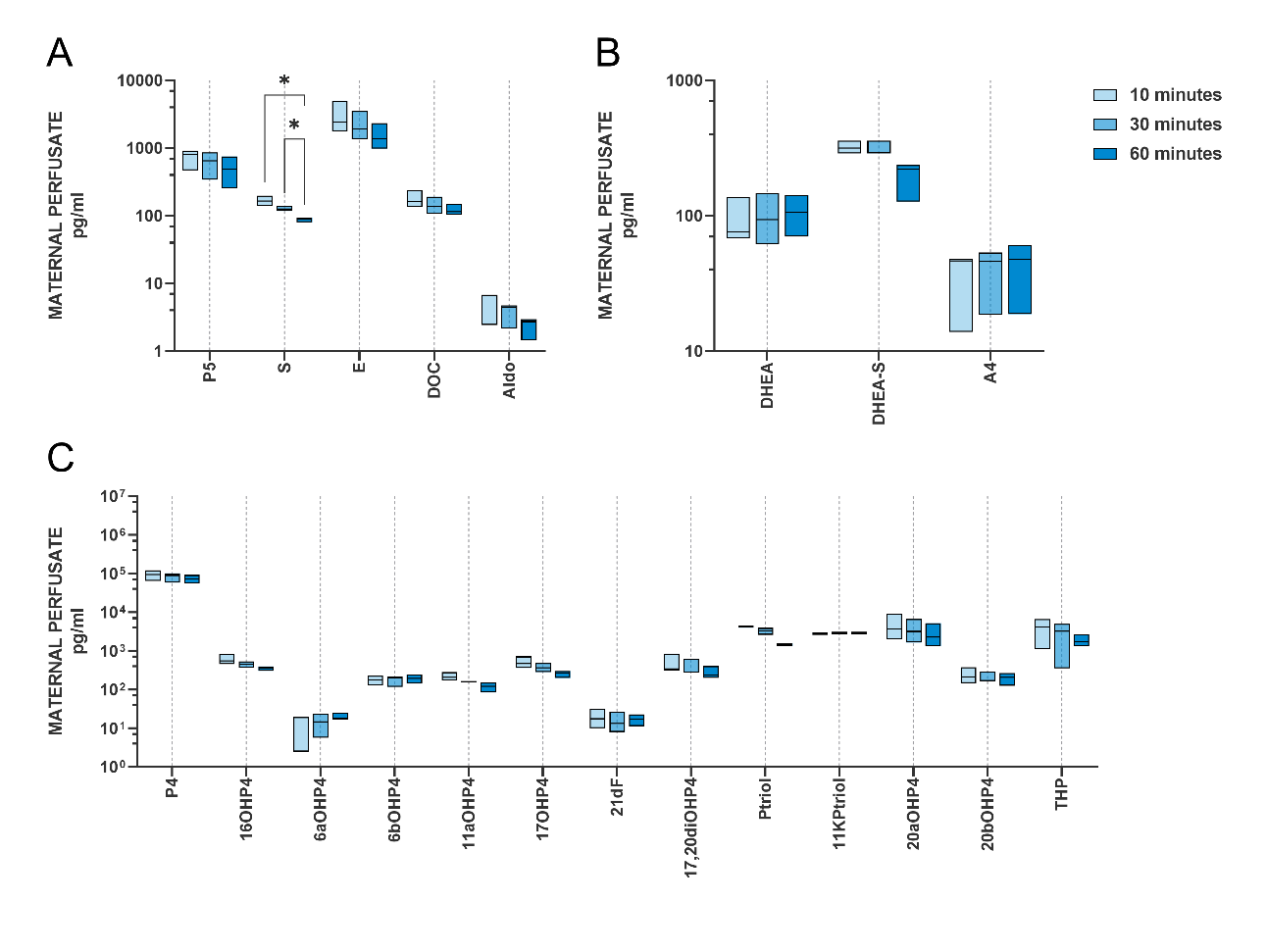


**Supplementary Figure 2.** Concentration of steroids in maternal venous perfusate. Steroids are categorized into mineralo-/glucocorticoids (A), androgens (B), and progesterones (C). The release of steroids in the maternal venous perfusate samples was evaluated at 10, 30, and 60 minutes. Data were log-transformed and are shown as boxplots; n = 3. Statistical analysis of time-dependent steroid release was evaluated using 2way ANOVA (p < 0,05*). Abbreviations of steroid species see Table 1 (manuscript file).

**
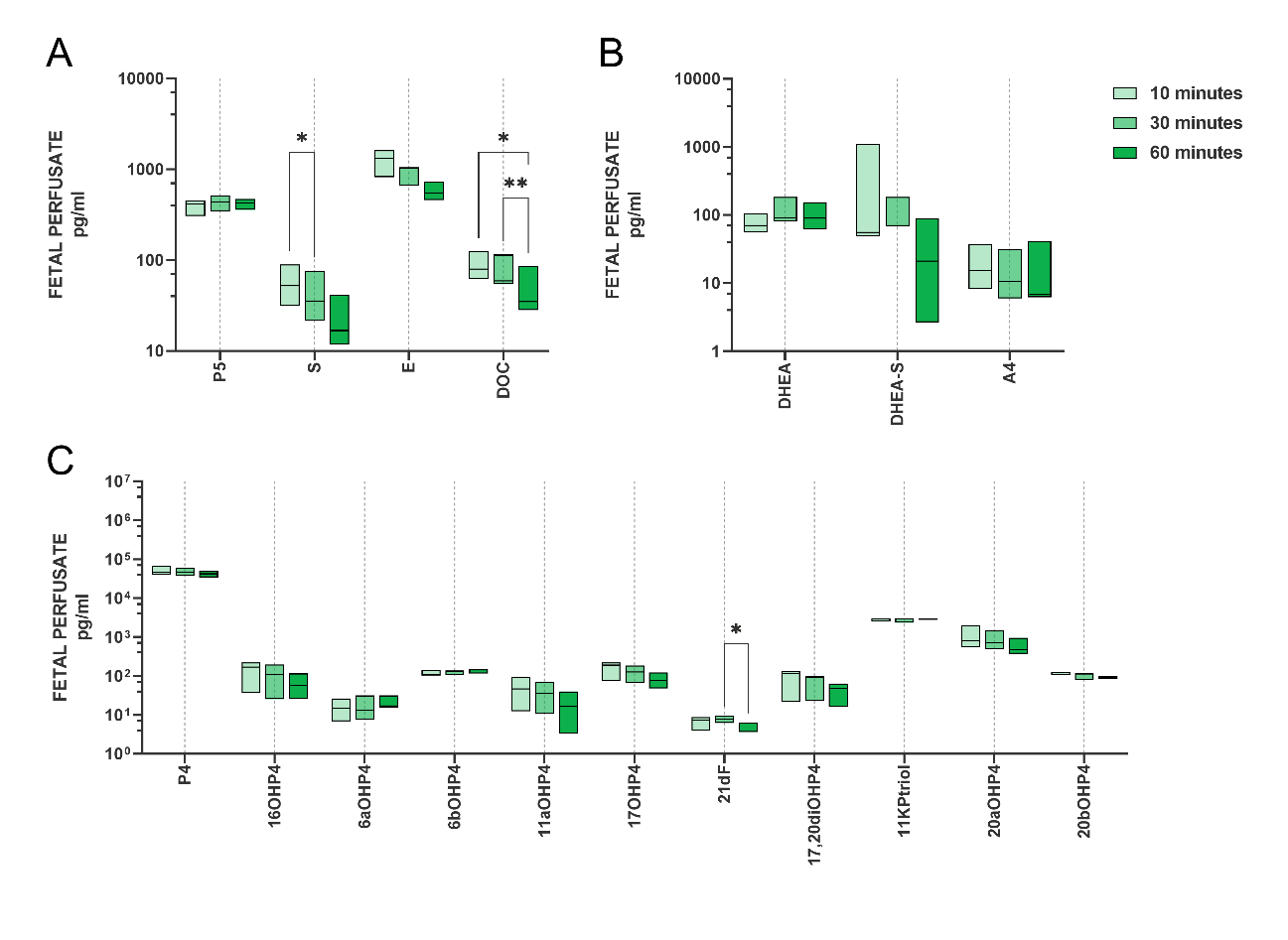
**

**Supplementary Figure 3.** Concentration of steroids in fetal venous perfusate. Steroids are categorized into mineralo-/glucocorticoids (A), androgens (B), and progesterones (C). The release of steroids in the fetal venous perfusate samples was evaluated at 10, 30, and 60 minutes. Data were log-transformed and are shown as boxplots; n = 3. Statistical analysis of time-dependent steroid release was evaluated using 2way ANOVA (p < 0,05*, p < 0,01**). Abbreviations of steroid species see Table 1 (manuscript file).

**
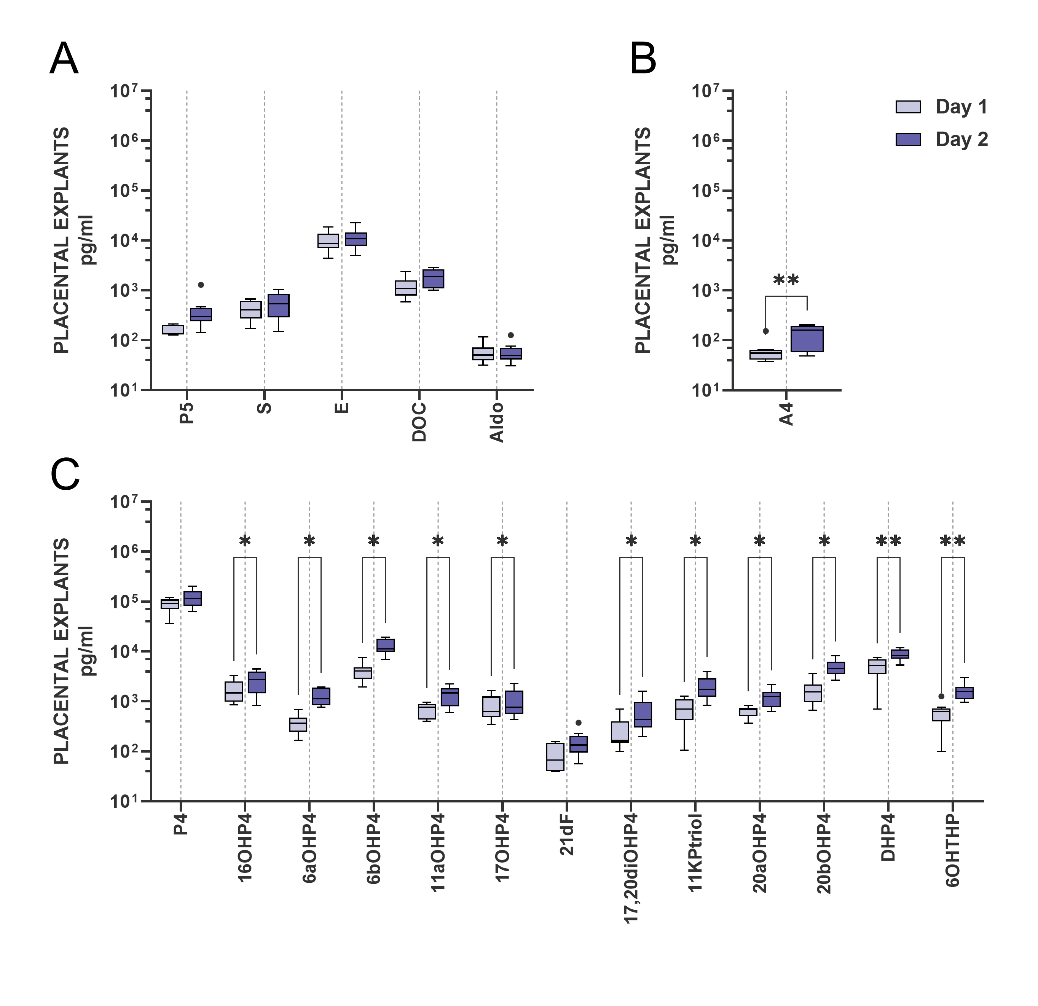
**

**Supplementary Figure 4.** Concentration of steroids in placental explants. Steroids are categorized into mineralo-/glucocorticoids (A), androgens (B), and progesterones (C). The release of steroids by human placental explants was evaluated after 24 hours (Day 1) and 48 hours (Day 2) of culturing. Data were log-transformed and are shown as boxplots; n = 9. Statistical analysis of time-dependent steroid release was evaluated using multiple paired t-tests (p < 0,05*, p < 0,01**). Abbreviations of steroid species see Table 1 (manuscript file).

**
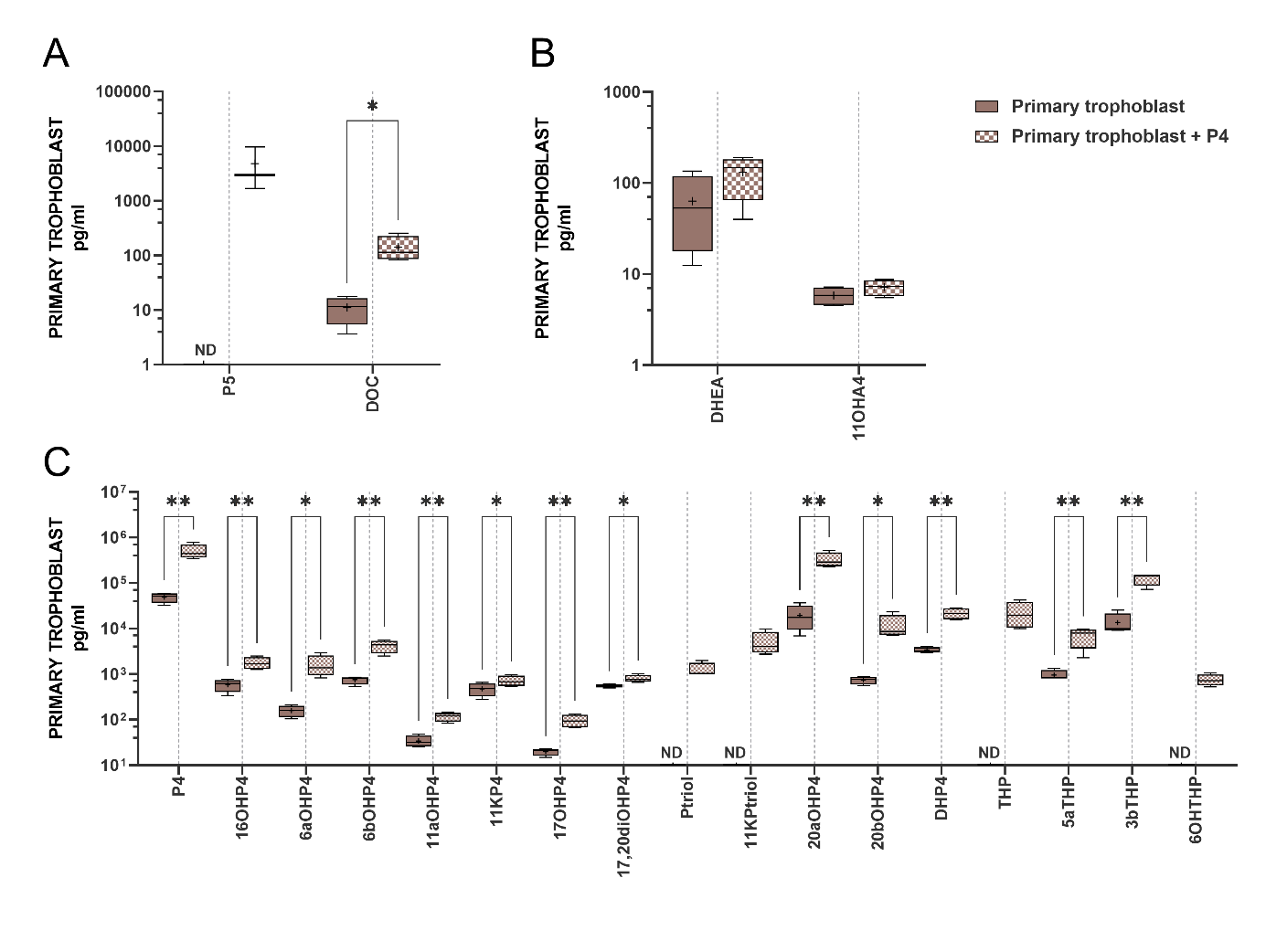
**

**Supplementary Figure 5.** Concentration of steroids in isolated primary trophoblast cells. Steroids are categorized into mineralo-/glucocorticoids (A), androgens (B), and progesterones (C). The release of steroids by human primary trophoblast cells was evaluated at basal state and upon spiking with 1 µM progesterone (P4). Data were log-transformed and are shown as boxplots; n = 4. Statistical analysis was evaluated using multiple paired t-tests (p < 0,05*, p < 0,01**). Abbreviations of steroid species see Table 1 (manuscript file).

**
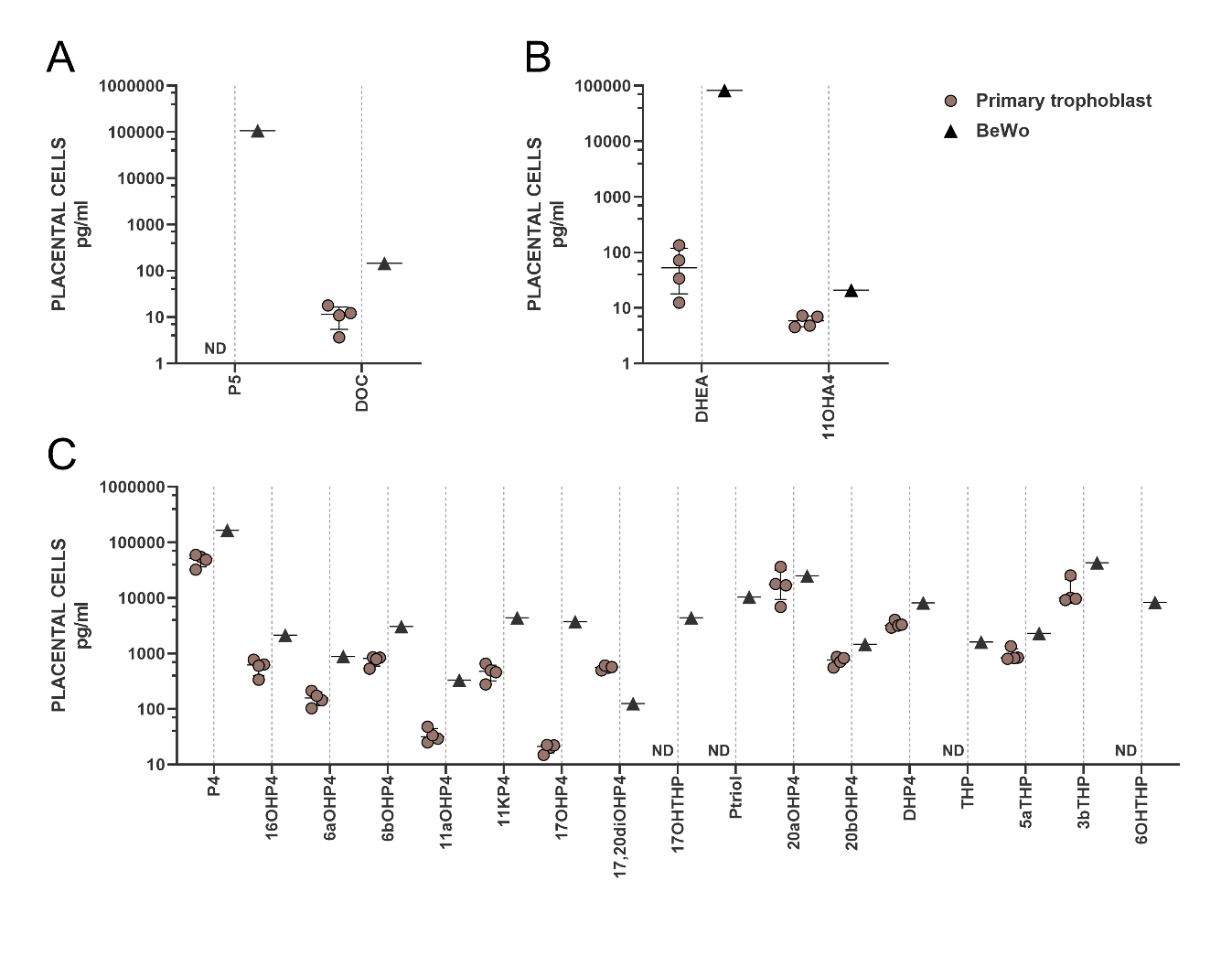
**

**Supplementary Figure 6.** Comparison of isolated primary trophoblast cells and the choriocarcinoma cell line BeWo in steroid release. Steroids are categorized into mineralo-/glucocorticoids (A), androgens (B), and progesterones (C). Data were log-transformed and are shown as individual data points; n = 4 (primary trophoblast cells) and n = 1 (BeWo cells). Steroids that were not detected (ND) are denoted in the graph. Abbreviations of steroid species see Table 1 (manuscript file).
